# Role of the DNA Mismatch Repair Gene *MutS4* in Driving the Evolution of *Mycobacterium yongonense* Type I via Homologous Recombination

**DOI:** 10.3389/fmicb.2017.02578

**Published:** 2017-12-20

**Authors:** Byoung-Jun Kim, Bo-Ram Kim, Yoon-Hoh Kook, Bum-Joon Kim

**Affiliations:** Department of Microbiology and Immunology, Biomedical Sciences, Liver Research Institute and Cancer Research Institute, College of Medicine, Seoul National University, Seoul, South Korea

**Keywords:** *Mycobacterium yongonense*, lateral gene transfer, DNA mismatch repair gene, MutS4, homologous recombination

## Abstract

We recently showed that *Mycobacterium yongonense* could be divided into two genotypes: Type I, in which the *rpoB* gene has been transferred from *Mycobacterium parascrofulaceum*, and Type II, in which the *rpoB* gene has not been transferred. Comparative genome analysis of three *M. yongonense* Type I, two *M. yongonense* Type II and *M. parascrofulaceum* type strains were performed in this study to gain insight into gene transfer from *M. parascrofulaceum* into *M. yongonense* Type I strains. We found two genome regions transferred from *M. parascrofulaceum*: one contained 3 consecutive genes, including the *rpoBC* operon, and the other contained 57 consecutive genes that had been transferred into *M. yongonense* Type I genomes via homologous recombination. Further comparison between the *M. yongonense* Type I and II genomes revealed that Type I, but not Type II has a distinct DNA mismatch repair gene (*MutS4* subfamily) that was possibly transferred via non-homologous recombination from other actinomycetes. We hypothesized that it could facilitate homologous recombination from the *M. parascrofulaceum* to the *M. yongonense* Type I genomes. We therefore generated recombinant *Mycobacterium smegmatis* containing a *MutS4* operon of *M. yongonense*. We found that the *M. tuberculosis rpoB* fragment with a rifampin resistance-conferring mutation was more frequently inserted into recombinant *M. smegmatis* than the wild type, suggesting that MutS4 is a driving force in the gene transfer from *M. parascrofulaceum* to *M. yongonense* Type I strains via homologous recombination. In conclusion, our data indicated that MutS4 in *M. yongonense* Type I genomes may drive gene transfer from *M. parascrofulaceum* via homologous recombination, resulting in division of *M. yongonense* into two genotypes, Type I and II.

## Introduction

Recombination is defined as process leading to the exchange of information between DNA or RNA and is a fundamental process with important implications for the evolution of the cell (Achtman and Wagner, 2008; Sheppard et al., 2008; Fraser et al., 2009). Recombination is typically classified as being homologous or non-homologous recombination, based upon the presence or absence of nucleotide sequence homology between the parental sites, respectively: homologous recombination, in which a fragment of a genome is replaced by one of sequence homology within another genome (Didelot and Maiden, 2010), and non-homologous recombination, which causes genetic additions and is often referred to as lateral gene transfer (LGT) (Ochman et al., 2000). Both homologous and non-homologous types of recombination are key elements in the evolution of bacteria and can be linked to variations in fitness and the consequent changes in ecologies and lifestyles (Didelot et al., 2012).

In most organisms, the mismatch repair system (MMR) pathway is highly conserved and enhances replication fidelity 50- to 1000-folds by repairing nucleotide mismatches and small insertions and deletions (Modrich and Lahue, 1996; Umar and Kunkel, 1996; Iyer et al., 2006). Members of the MutS and MutL protein families normally play a pivotal role in mismatch correction. The MMR system also prevents recombination between not identical DNA sequences (homeologous recombination) (Reenan and Kolodner, 1992). Defects in the MMR system could therefore lead to highly elevated mutation rates (hypermutability), meiotic defects and infertility (Harfe and Jinks-Robertson, 2000; Surtees et al., 2004). The genus *Mycobacterium* has no homologs of MutS or MutL (Mizrahi and Andersen, 1998; Sachadyn, 2010; Banasik and Sachadyn, 2014). Instead, its genome stability is maintained via an alternative NucS pathway that appears in many Archaea (Castaneda-Garcia et al., 2017).

Homologous recombination and homeologous recombination are important mechanisms that contribute to the genomic diversity of various bacteria. To restrict recombination between moderately divergent (up to ∼10%) DNA sequences at the DNA hybridization step, prokaryotes and eukaryotes utilize a canonical MutS–MutL-based MMR system that facilitates gene transfer via homologous recombination during eukaryotic meiosis in eukaryotes or during genome acquisition from foreign bacterial DNA (Modrich and Lahue, 1996; Vulic et al., 1997). Previous reports that genes acquired from other bacteria are rarely found in the genomes of *M. tuberculosis* or *M. leprae*, both lacking the highly conserved MutS-based MMR system (Vulic et al., 1997; Cole et al., 1998, 2001), strongly support the above notion.

We recently found that *Mycobacterium yongonense* can be divided into two genotypes: Type I, in which the *rpoB* gene has been transferred from *Mycobacterium parascrofulaceum*, and Type II, in which the *rpoB* gene has not been a subject of gene transfer (Kim et al., 2016). Comparative genome analysis between three *M. yongonense* Type I (DSM 45126^T^, MOTT-12 and MOTT-27) and two *M. yongonense* Type II (MOTT-36Y and MOTT-H4Y) strains and an *M. parascrofulaceum* type strain (ATCC BAA-614^T^) was performed to gain insight into the gene transfer from *M. parascrofulaceum* to *M. yongonense* Type I strains. We found for the first time in mycobacteria a distinct DNA mismatch repair gene that belonged to the *MutS4* subfamily in the genome of *M. yongonense* Type I strains and that served as a putative driving force of homologous recombination between the *M. parascrofulaceum* and *M. yongonense* Type I genomes.

## Results

### Identification of Two Putative Regions in the *M. yongonense* Type I Genome That Were Transferred from *M. parascrofulaceum*

**FIGURE 1:**
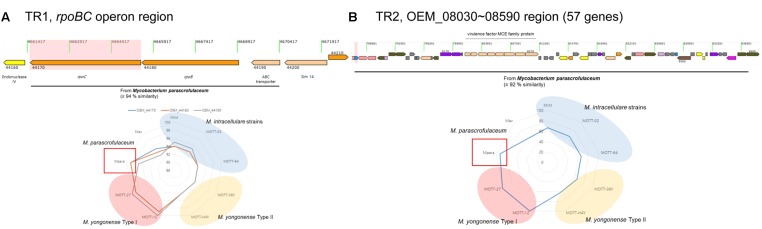
Visualization of two putative lateral gene transferred regions within the *M. yongonense* DSM 45126^T^ genome. **(A)** First region (*rpoBC* operon, OEM_44170∼44190) (TR1). **(B)** Second region (57 ORFs, OEM_08030∼08590) (TR2). Sequence similarities between *M. yongonense* DSM 45126^T^ and related strains were also visualized in each lower image. Red region, *M. yongonense* Type II strains; yellow region, *M. yongonense* Type I strains; blue region, *M. intracellulare*.

As described in a previous report, *M. yongonense* Type I strains (DSM 45126^T^, MOTT-12 and MOTT-27) have an *rpoB* gene that may have been transferred from the distantly related scotochromogenic species *M. parascrofulaceum* (Kim et al., 2013a,b,c, 2016). The *rpoB* gene was also found to differ between the *M. yongonense* Type I (DSM 45126^T^, MOTT-12 and MOTT-27) and Type II strains (MOTT-36Y and MOTT-H4Y) used in this study. The complete genome sequences of two *M. yongonense* Type I strains, MOTT-12 (GenBank accession No, CP015964) and MOTT-27 (GenBank accession No, CP015965), were analyzed in this study to gain further insight into gene transfer from *M. parascrofulaceum* to *M. yongonense* Type I (**Table 1**). All the ORFs of seven strains (three *M. yongonense* Type I strains, two *M. yongonense* Type II, strains and one *M. parascrofulaceum* strain) were compared and analyzed using the BLASTN and BLASTP programs. Two loci that showed higher sequence similarities to sequences in *M. parascrofulaceum* than to those in the phylogenetically related *M. yongonense* Type II strains were found in the genomes of the three *M. yongonense* Type I strains. The first region includes three consecutive ORFs, an ABC transporter and the *rpoB* and *rpoC* genes [designated as TR1 (Transfer Region 1), OEM_44170∼44190 in *M. yongonense* DSM 45126^T^], and the second region contains 57 consecutive ORFs, including genes corresponding to dehydrogenase, MCE family, which could enable mycobacteria to enter into and survive inside the mammalian macrophage (Arruda et al., 1993; Kumar et al., 2003; Zhang and Xie, 2011), and fatty acid biosynthesis [designated as TR2 (Transfer Region 2), OEM_08030∼08590 in *M. yongonense* DSM 45126^T^] (**Figure 1** and Supplementary Table S1). All 60 transferred ORFs of the *M. yongonense* Type I strains (DMS 45126^T^, MOTT-12, and MOTT-27) were always more closely related to sequences in *M. parascrofulaceum* than those in *M. intracellulare* and *M. yongonense* Type II strains [in TR1 (3 ORFs), sequences were 97–99% similar to their counterparts in *M. parascrofulaceum*, and in TR2 (57 ORFs), sequences were 95–100% similar to their counterparts in *M. parascrofulaceum*] (**Figure 1** and Supplementary Table S1). The ABC transporter (OEM_44190) and *rpoC* (OEM_44170), which correspond to the two ends (5′ and 3′) of TR1, of *M. yongonense* Type I strains (DSM 45126^T^, MOTT-12 and MOTT-27) were clustered with their homologs from *M. parascrofulaceum* (92 or 100% bootstrap values) (Supplementary Figures S1B,C). However, the outer neighboring ORFs (OEM_44200; sim14 and OEM_44160; endonuclease IV) of *M. yongonense* Type I strains were more closely grouped with those of *M. intracellulare* (ATCC 13950^T^, MOTT-02 and MOTT-64) or *M. yongonense* Type II (MOTT-36Y and MOTT-H4Y) strains than those of the *M. parascrofulaceum* strain (Supplementary Figures S1A,D). A similar trend was also found in TR2 (Supplementary Figure S2).

**Table 1:** Genome sequences used in this study.

Strains	GenBank accession no.	Genome size (bp)	G+C ratio (%)	CDS	tRNA	INT-group	Reference
*M. intracellulare* ATCC 13950^T^	CP003322	5,402,402	68.10	5,145	47	INT-2	Forrellad et al., 2013
*M. intracellulare* MOTT-02	CP003323	5,409,696	68.10	5,151	47	INT-2	Kim et al., 2015
*M. intracellulare* MOTT-64	CP003324	5,501,090	68.07	5,251	46	INT-1	Kim et al., 2012c
*M. yongonense* DSM 45126^T^	CP003347	5,521,023	67.95	5,222	47	INT-5	Kim et al., 2012d
*M. yongonense* MOTT-12	CP015964	5,445,538	68.02	5,157	47	INT-5	In this study
*M. yongonense* MOTT-27	CP015965	5,435,152	68.03	5,041	47	INT-5	In this study
*M. yongonense* MOTT-36Y	CP003491	5,613,626	67.91	5,128	46	INT-5	Kim et al., 2012b
*M. yongonense* MOTT-H4Y	AKIG00000000	5,443,025	68.08	5,020	48	INT-5	Kim et al., 2013b
*M. avium* 104	NC_008595	5,475,491	68.99	5,120	46	–	–
*M. parascrofulaceum* ATCC BAA-614^T^	ADNV00000000	6,564,170	68.5	5,586	47	–	–

### Identification of Homologous Recombination Sites in the Two Putative Transferred Regions, TR1 and TR2 of the *M. yongonense* Type I Genome

**FIGURE 2:**
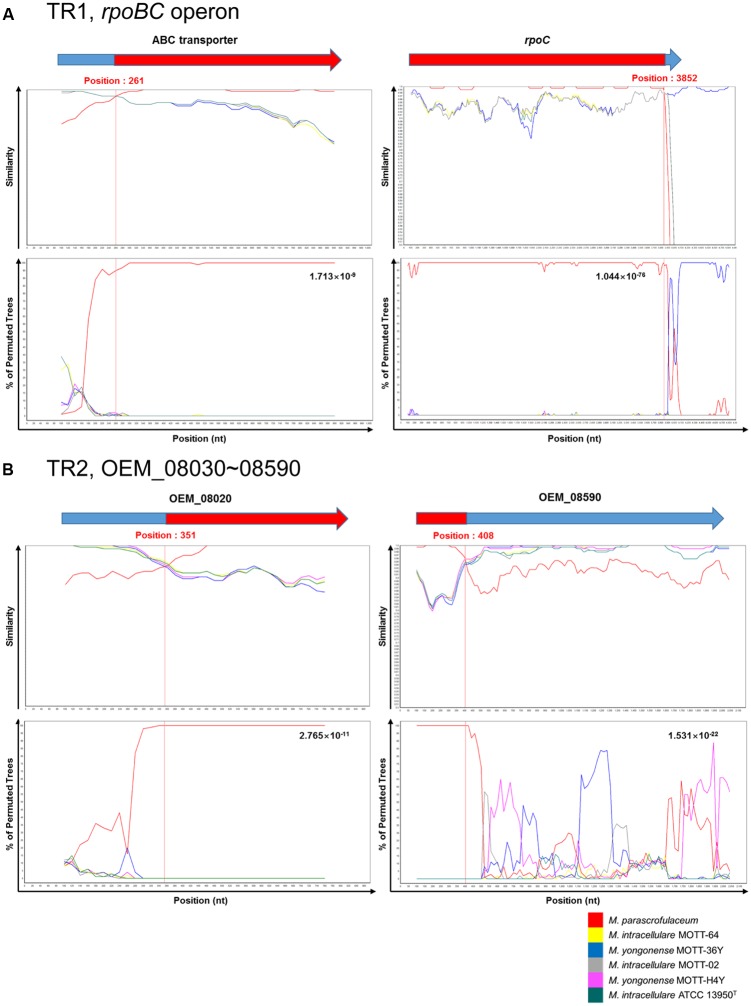
Plots of similarity in a putative recombination site from *M. intracellulare* (ATCC 13950^T^, MOTT-02 and MOTT-64) or *M. yongonense* Type II (MOTT-36Y and MOTT-H4Y) and *M. parascrofulaceum* strains to *M. yongonense* Type I (DSM 45126^T^, MOTT-12 and MOTT-27) strains. **(A)** First recombination site (from OEM_44170 to 44190) (TR1). **(B)** Second recombination site (from OEM_08030 to 08590) (TR2). Each figure indicate SimPlot and BootScan results of *M. yongonense* Type I strains compared to *M. intracellulare*, *M. yongonense* Type II and *M. parascrofulaceum* strains. Each point plotted is percent identity within a sliding window 200 bp wide centered on the position plotted, with a 20-bp step size between points. Detailed parameters used for analysis are as follows; Window: 200 bp, Step: 20 bp, GapStrip: On, Reps: 100, Kimura (2-parameter), T/t: 2.0, Neighbor-joining.

To identify potential breakpoints for gene transfer within the two putative transferred regions, TR1 and TR2, of the *M. yongonense* Type I genome, we applied BootScan analysis to the TR1 and TR2 sequences of nine mycobacterial strains (three *M. yongonense* Type I, two *M. yongonense* Type II and *M. parascrofulaceum* and *M. intracellulare* type strains). Potential locations of the recombination breakpoints of the 5′ and 3′ ends were found in TR1 at the 261st nucleotide (nt) of OEM_44190 (ABC transporter) and the 3,852nd nt of OEM_44170 (*rpoC*), respectively. Aligned sequences showed 44-bp sequences flanking the potential breakpoints of the 5′ end (nt 229–272 of the ABC transporter) and 26-bp sequences flanking the potential breakpoints of the 3′ end (nt 3920–3945 of *rpoC*), which were conserved in all nine aligned mycobacterial strains. The aligned and phylogenetic profiles clearly differed in the sequences located at the 5′ and 3′ ends of these conserved regions. The potential locations of the recombination breakpoints of the 5′ and 3′ ends in TR2 were found at the 351st nt of OEM_08020 and the 408th nt of OEM_08590, respectively (**Figure 2** and Supplementary Figure S3). Aligned sequences showed 29-bp sequences flanking the potential breakpoints of the 5′ end (nt 337–365 of OEM_08020) and 34-bp sequences flanking the potential breakpoints of the 3′ end (nt 410–443 of OEM_08590), which were conserved in almost all seven aligned mycobacterial strains despite minor differences. Our data suggest that TR1 and TR2 of the *M. yongonense* Type I genome may have been transferred from *M. parascrofulaceum* via homologous recombination.

### Identification of Distinct *MutS4*-Related DNA Mismatch Repair Genes in the Genome of *M. yongonense* Type I Strains

**FIGURE 3:**
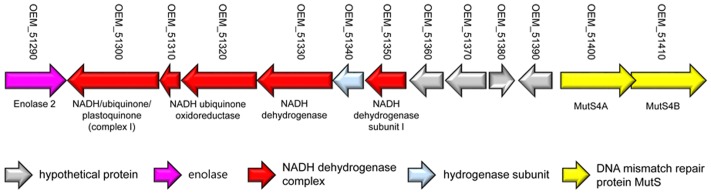
Presentation of a locus containing non-mycobacterial genes in *M. yongonense* Type I strains, but not Type II strains.

**FIGURE 4:**
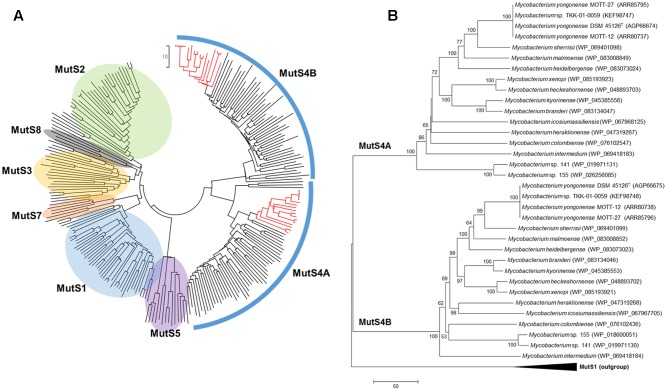
Neighbor-Joining phylogenetic trees of MutS family proteins. **(A)** Phylogenetic tree covering MutS homologs from diverse bacterial and archaeal strains. Red branches indicate MutS4 homologs of *Mycobacterium* strains including *M. yongonense* Type I strains. **(B)** Phylogenetic tree based on MutS4 homologs among *Mycobacterium* strains and rooted with MutS1 sequences as an out-group. Evolutionary distances are in units of numbers of amino acid differences per sequence.

Although *M. yongonense* Type I and Type II strains are members of the same species, only *M. yongonense* Type I strains have unique gene regions, TR1 and TR2, that were transferred from *M. parascrofulaceum* via homologous recombination. This finding prompted us to hypothesize that there may be distinct ORFs in the *M. yongonense* Type I genome that drive gene acquisition via homologous recombination. To address this issue, we analyzed the putative genomic islands in the *M. yongonense* genome by web-based program, “IslandViewer 4”^1^ (Didelot and Maiden, 2010; Zhang and Xie, 2011). The result showed that the 9 putative genomic islands were identified from genome of the *M. yongonense* Type I (DSM 45126^T^) (Supplementary Figure S4). Among these putative genomic islands, a distinct region composed of 13 consecutive ORFs that was possibly transferred from non-mycobacterial actinomycetes was found in the genome of only *M. yongonense* Type I strains (DSM 45126^T^, MOTT-12 and MOTT-27), but not in the genome of *M. yongonense* Type II strains (MOTT-36Y and MOTT-H4Y) (**Figure 3**, Supplementary Table S2, and Supplementary Figure S5). This region contains enolase (OEM_51290), NADH dehydrogenase complex (OEM_51300∼51330 and 51350), hydrogenase subunit (OEM_51340) and DNA mismatch repair genes (OEM_51400 and 51410) (**Figure 3**, Supplementary Table S2, and Supplementary Figure S5). Since the DNA mismatch repair gene has been reported to result from homologous recombination (Lin et al., 2007), we hypothesized that the two consecutive ORFs (OEM_51400 and 51410) encoding DNA mismatch repair genes, which are distinct in *M. yongonense* Type I strains, could drive gene transfer from *M. parascrofulaceum* to *M. yongonense* Type I strains via homologous recombination. Notably, the two ORFs of the DNA mismatch repair genes were MutS4A and MutS4B homologs of the *MutS4* subfamily (**Figure 4**). Furthermore, detailed sequence inspection showed that these two ORFs (OEM_51400 and 51410) also have a signature structure from the *MutS4* subfamily; *MutS4A* and *MutS4B* are adjacent, and the stop codon of *MutS4A* overlaps with the initiation codon of *MutS4B* (Supplementary Figure S6).

### Phylogenetic Analysis of Mycobacterial MutS4 Orthologs

To confirm the presence of *MutS4* genes in mycobacterial species other than *M. yongonense* Type I strains, the amino acid sequences of *M. yongonense* Type I MutS4 were subject to BLAST analysis against mycobacterial genome databases. We found MutS4 orthologs in another 14 of the 109 mycobacterial species whose complete or draft genomes have been introduced (Supplementary Table S3). We confirmed that all 14 strains have two MutS4 homologs, MutS4A and MutS4B, that share the signature structure of the MutS4 subfamily. Global phylogenetic analysis using the MutS amino acid sequences of eubacteria, archaebacteria and eukaryotes showed that 17 mycobacteria were located in a distinct cluster based on similarities in MutS4A or MutS4B (**Figure 4**). A difference in mycobacterial phylogenetic topology between MutS4A and MutS4B was not observed. Notably, two MutS4 ORFs of the *Mycobacterium* sp. TKK-01-0059 strain isolated from Ngwelezane, South Africa shared 100% sequence similarities with those of *M. yongonense* Type I strains, suggesting that this strain may be a member of the *M. yongonense* Type I family. Further *hsp65* and genome sequence-based phylogenetic analyses also support this hypothesis (Supplementary Figures S7, S8).

To address the origin of mycobacterial *MutS4* genes and determine whether they were present because of an LGT mechanism, we compared the topology between the phylogenetic trees of *MutS4* genes, the *hsp65* as a chronometer gene and whole genome sequences. Incongruence between the tree topologies of MutS4 and *hsp65* or whole genome sequences was found. For example, *M. colombiense*, a member of the *Mycobacterium avium* complex (MAC), was the closest related to *Mycobacterium intermedium* (MutS4A tree) or to *Mycobacterium* sp. 141 and 155 strains (MutS4B tree) in MutS4 based trees; but, this species was closely located to *M. yongonense* strains both in the *hsp65*-based tree and in the whole genome-based tree (Supplementary Figures S7, S8). This result strongly supports an evolutionary scenario that includes the distribution of the *MutS4* gene into several mycobacterial species via LGT.

### Increased Frequency of Homologous Recombination in Recombinant *M. smegmatis* Harboring a *M. yongonense* Type I *MutS4* Operon

**FIGURE 5:**
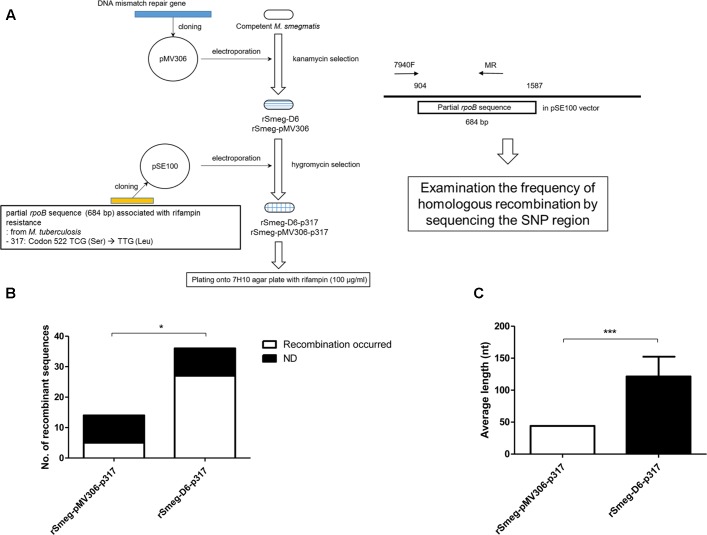
Analysis the putative homologous recombination event in recombinant *M. smegmatis* harboring DNA mismatch repair genes and partial *rpoB* gene of *M. tuberculosis*. **(A)** Schematic flow for analyzing frequency of homologous recombination using recombinant *M. smegmatis* harboring DNA mismatch repair genes and partial *rpoB* gene of *M. tuberculosis*. **(B)** Comparison the numbers of putative homologous recombined *rpoB* sequences in the transformed recombinant strains which were grown on 7H10 agar plate supplemented with rifampin. From the transformed colonies, partial *rpoB* was analyzed whether the sequence was recombined with rifampin resistant *M. tuberculosis* partial *rpoB* gene sequence. ND means the colonies where recombination has not been occurred. *P*-value was calculated by Chi-square test. ^∗^*P* < 0.05. **(C)** Comparison the average length of putative homologous recombined sequences in the transformed recombinant *M. smegmatis* strains. *P*-value was calculated by student’s-*t* test. ^∗∗∗^*P* < 0.001.

To examine the role of *M. yongonense* Type I MutS4 in homologous recombination, we amplified the region (3,838 bp) including *MutS4A* (OEM_51400), *MutS4B* (OEM_51410) and their promoter from a *M. yongonense* Type I strain (DSM 45126^T^) as described in the Methods (Supplementary Figure S6). This amplicon was cloned into the integrative pMV306 vector and transformed into *M. smegmatis* to generate a recombinant *M. smegmatis* harboring a *M. yongonense* Type I *MutS4* operon or an empty vector (rSmeg-D6 or rSmeg-pMV306). The recombinant *M. smegmatis* strains were confirmed by colony PCR and RT-PCR (Supplementary Figure S9). To confirm the role of the *MutS4* operon in homologous recombination, we created a pSE100-317 vector with a *M. tuberculosis* partial *rpoB* sequence (684 bp) containing a mutation in codon 522 (TCG → TTG; 317); this mutation confers resistance to rifampin. The constructed pSE100-317 was then transformed into rSmeg-D6 or rSmeg-pMV306 (rSmeg-D6-p317 or rSmeg-pMV306-p317) (**Figure 5A**). After the transformed strains were plated onto 7H10 agar medium with 100 μg/ml of rifampin. Colonies grown on the rifampin medium (100 μg/ml) were judged as potential recombinants and final authenticity of their recombination were confirmed via checking the presence of *M. tuberculosis* specific SNPs related to rifampin resistance (mutation at codon 522) by PCR-sequencing protocol targeting the *rpoB* region. Also, the break point between the *M. smegmatis*-distinct and *M. tuberculosis*-distinct sequences was considered to be a potential recombination site. From the three independent trials, total of 22 (rSmeg-pMV306-p317; 7.33 ± 2.52 colonies/trial) and 55 (rSmeg-D6-p317; 18.33 ± 3.06 colonies/trial) colonies were grown on the rifampin medium and identified as putative recombinants. Among them, 16 colonies of rSmeg-pMV306-p317 and 36 colonies of rSmeg-D6-p317 were randomly selected and used for sequencing the *rpoB* region. Sequence analysis of rSmeg-D6-p317 showed that 27 colonies (75%) of 36 selected colonies grown in rifampin 7H10 agar have a SNP at codon 522 (TCG → TTG) that confers resistance to rifampin. In the case of rSmeg-pMV306-p317, from the 16 selected colonies, only five colonies (31.3%) have changed SNP at codon 522 (**Figure 5B**, Supplementary Figure S10, and Supplementary Table S4). This result means that the rifampin resistance of these colonies is due to the recombined resistance-conferring *M. tuberculosis rpoB* gene and is not induced by the mutation of the *M. smegmatis rpoB*. The average length of the recombined *M. tuberculosis rpoB* gene in rSmeg-D6-p317 strains (121.3 ± 31.0 nt) is significantly longer than that in the control strain, rSmeg-pMV306-p317 (44.0 ± 0.0 nt) (**Figure 5C** and Supplementary Table S4), suggesting that the *MutS4* gene of *M. yongonense* Type I strains plays a pivotal role in homologous recombination in *M. smegmatis*.

## Discussion

In this study, we found the first MutS homologs in *Mycobacterium* genomes via genome analysis of three *M. yongonense* Type I strains (**Figures 3**, **4**). Most unexpectedly, our BLAST analysis indicated that another 14 of 109 mycobacterial species whose whole genomes are currently available, also have MutS4 orthologs. Notably, all 17 mycobacterial strains with MutS4, including the three *M. yongonense* type I strains, are slowly and not rapidly growing mycobacteria (**Figure 4B** and Supplementary Table S3). However, the incongruence between phylogenetic analyses based on MutS4 homologs and the *hsp65* gene or whole genome sequences, strongly supports our hypothesis that MutS4 distribution between slow-growing mycobacterial strains may also be due to LGT (**Figure 4B** and Supplementary Figures S7, S8). For example, both *M. yongonense* and *M. colombiense* belong to members of the same MAC (Kim et al., 2013c). However, they are phylogenetically separated in *MutS4* gene-based phylogenetic analysis, suggesting that the acquisition of the *MutS* gene may have recently occurred via LGT. *MutS4* is present only in several distantly related bacter4ial species (Lin et al., 2007), and most strains contain two copies, *MutS4A* and *MutS4B*, whose sequences are phylogenetically closely related to each other, suggesting their generation by duplication in an ancestral bacterial strain (Lin et al., 2007). The signature gene structure present between *MutS4A* and *MutS4B* in a bacterial genome was also found, i.e., they are adjacent and the stop codon of *MutS4A* overlaps with the initiation codon of *MutS4B* (Lin et al., 2007). We also confirmed that all mycobacterial strains contain two copies, *MutS4A* and *MutS4B*, and have the conserved signature gene structure in their genomes.

**FIGURE 6:**
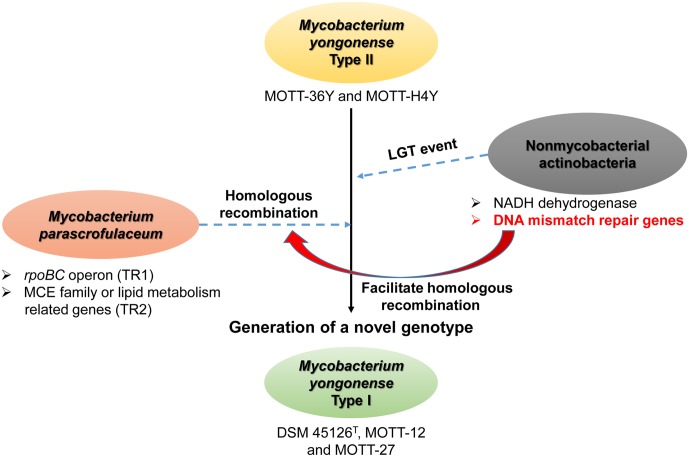
Schematic representation showing role of *MutS4* gene in evolution of *M. yongonense* Type I via homologous recombination event of TR1 and TR2 from *M. parascrofulaceum* to *M. yongonense* Type I.

A *MutSac* domain in the *MutS4* gene is expected to be involved in yet to be defined functions related to DNA metabolism in bacteria (Lin et al., 2007). However, its absence from most bacteria suggests that its functions are not essential and are gradually lost during evolution (Lin et al., 2007). Nevertheless, recent acquisition of MutS4 homologs by several mycobacterial species during evolution is more or less unusual. Two different genotypes of *M. yongonense* differ in the presence of a MutS4 homolog, leading to speculating that comparative genome analysis of Type I and II strains can provide a clue to the putative role of the *MutS4* gene in mycobacterial evolution (**Figure 6**). In fact, our detailed inspection of genome sequences indicated that there are three distinctly different regions between the genomes of two different genotypes of *M. yongonense*, Type I and Type II. The first region is present only in the genome of Type I strains, not that of Type II, and includes 13 consecutive ORFs (OEM_51290 to 51410) (**Figure 3** and Supplementary Table S2), including a *MutS4* gene that may have been acquired from non-mycobacterial actinobacteria via LGT (**Figure 6**). Of these genes, the enolase-coding ORF (OEM 51290) and six consecutive ORFs related to the NADH dehydrogenase complex (OEM 51290-51350) appear to be related to mycobacterial pathogenesis (Velmurugan et al., 2007; Miller et al., 2010). The second and third regions, which are also distinct in the Type I genome, consists of three consecutive genes, including the *rpoBC* operon (OEM_44170 to 44190) (TR1) (**Figure 1A** and Supplementary Table S1), and 57 consecutive genes (OEM_08030 to 08590) (TR2) (**Figure 1B** and Supplementary Table S1), respectively, that may have been acquired from *M. parascrofulaceum* via homologous recombination, respectively. We hypothesized that the LGT of *MutS4* to an ancestor of *M. yongonense* Type I strains facilitated the transfer of approximately 60 genes from *M. parascrofulaceum* into the genome of *M. yongonense* by a homologous recombination mechanism, leading to distinct evolutionary pathways between *M. yongonense* Type I and Type II strains. Indeed, we found that rSmeg reinforced by the *MutS4* operon of *M. yongonense* Type I strain exhibited a significantly more frequent homologous recombination when transformed with an *M. tuberculosis rpoB* fragment carrying a rifampin-resistance (*rif^R^*)-related mutation than *M. smegmatis* reinforced by a mock plasmid (pMV306 only) (**Figures 5B,C**, Supplementary Figure S10, and Supplementary Table S4), suggesting that the *MutS4* gene plays a central role in gene transfer by homologous recombination in mycobacteria.

Genetic exchanges in the *rpoB* gene between closely related subspecies within the *M. abscessus* complex were recently reported to frequently occur (Macheras et al., 2011; Kim et al., 2017). In terms of clinical diagnostics, the *rpoB* typing method lacks power, culminating in 20% failure rates in the *M. massiliense* subspecies (Sapriel et al., 2016) and thus suggesting they have hybrid *rpoB* genes, a part of which were from *M. abscessus* subspecies. Since only a small portion of *rpoB* in the *M. abscessus* complex is exchanged between the same species, the function of the resulting hybrid products may be almost the same as that of the original. However, *M. yongonense* Type I strains differ because almost all of their *rpoBC* operon (but not its major interacting partners, such as *rpoA* and *rpoD*) are transferred from a distantly related species, *M. parascrofulaceum* (data not shown). This may be the first report of LGT of the entire *rpoBC* operon. A comparison of the deduced amino acid sequences showed a marked difference in the *rpoBC* operons of *M. yongonense* Type I and II strains, possibly indicating changes in function. The issue of evolutionary merit in the LGT of *M. parascrofulaceum rpoBC* to the *M. yongonense* Type I genome remains a mystery. No significant difference in rifampin resistance between *M. yongonense* Type I and II strains was found (data not shown). One plausible explanation is that transferred *M. parascrofulaceum rpoBC* contributed to the facilitated expression of the 57 consecutive genes (OEM_08030 to 08590) in TR2 that were acquired in *M. yongonense* Type I strains from *M. parascrofulaceum* via homologous recombination.

TR2, within the *M. yongonense* Type I genome, was composed of 57 consecutive genes (OEM_08030 to 08590), was putatively transferred from *M. parascrofulaceum* and includes several virulence-related *mce* family genes and fatty acid biosynthesis-related genes capable of affecting mycobacterial cell wall structure, possibly leading to a change in host-pathogen interactions (Zhang and Xie, 2011; Forrellad et al., 2013). Phylogenetic analysis of every single gene indicated that 53 of the 57 consecutive genes within TR2 closely clustered with *M. parascrofulaceum* but not *M. yongonense* Type II genes (**Figure 1B** and Supplementary Table S1), with most showing 100% sequence similarity with their *M. parascrofulaceum* orthologs. The four genes not clustered into *M. parascrofulaceum* (OEM_08190, OEM_08520 to 08540) have no corresponding orthologs in the *M. parascrofulaceum* or *M. yongonense* Type II genomes, suggesting that their presence may be due to intragenomic gene transfer by mobile genetic elements within the *M. yongonense* Type I strain’s own genome, rather than to gene loss followed by gene transfer from *M. parascrofulaceum*. Indeed, the three consecutive genes (OEM_08520 to 08540) proved to be insertion sequence (IS) elements that are frequently reported to be found in *M. yongonense* (Kim et al., 2015), and supporting the above hypothesis.

In conclusion, our genome sequence-based phylogenetic analysis and gain-of-function experiment using rSmeg indicated that the *MutS4* gene of *M. yongonense* Type I could play a pivotal role in mycobacterial evolution via increasing genetic transfer through homologous recombination from other distantly related mycobacteria.

## Materials and Methods

### Genome Sequences Used in This Study

Ten mycobacterial genome sequences, from strains belonging to the *M. avium* complex [3 *M. intracellulare* (Mint) strains: ATCC 13950^T^, MOTT-02, and MOTT-64; 5 *M. yongonense* strains: DSM 45126^T^, MOTT-12, -27, -36Y and -H4Y; one *M. avium* (Mav) strain: *M. avium* 104; and one *M. parascrofulaceum* strain: *M. parascrofulaceum* ATCC BAA-614^T^] (Kim et al., 2012a,b,c,d, 2013b; Lee et al., 2013) and were retrieved from the GenBank database (**Table 1**) and used for comparative genome analysis.

### Identification of Putative Lateral Gene Transferred Regions in *M. yongonense* Type I Strains from *M. parascrofulaceum* or Other Genus Strains

To identify putative lateral gene transferred regions of *M. yongonense*, all ORFs were compared and analyzed using BLASTN and BLASTP programs. The ORFs with high sequence similarities to *M. parascrofulaceum* (compared length > 80% and sequence similarities > 80% in nucleotide sequence) were selected and analyzed for possible recombination events. Also, webserver based program, IslandViewer 4^2^ (Langille and Brinkman, 2009; Bertelli et al., 2017) was used to identify and visualize the putative genomic islands in the genome of *M. yongonense* DSM 45126^T^ with three prediction methods: IslandPick (Langille et al., 2008), IslandPath (Hsiao et al., 2003) and SIGI-HMM (Langille and Brinkman, 2009). Among the selected putative regions, ORFs with high sequence similarities to other genus in the genome of *M. yongonense* DMS 45126^T^ (*M. yongonense* Type I) were selected and compared to two other *M. yongonense* Type II (MOTT-36Y and MOTT-H4Y), three *M. intracellulare* (ATCC 13950^T^, MOTT-02 and MOTT-64) and *M. parascrofulaceum* strains. Finally, ORFs that are specific for *M. yongonense* Type I strains, and not found in other comparative strains were identified, multiply aligned and visualized by Mauve multiple genome alignment system.^3^

### Construction of Phylogenetic Trees and SimPlot Analysis

All identified ORFs from the genome of *M. yongonense* DSM 45126^T^ were compared to other *M. intraceullulare* (ATCC 13950^T^, MOTT-02 and -64), *M. yongonense* Type I (MOTT-12 and -27), *M. yongonense* Type II (MOTT-36Y and -H4Y), *M. avium* and *M. parascrofulaceum* strains (**Table 1**). MutS proteins in the *M. yongonense* Type I strains (DSM 45126^T^, MOTT-12, and -27) were compared to MutS homologs from other bacteria or viruses (Lin et al., 2007; Ogata et al., 2011) and additional MutS4 family sequences which were retrieved from the GenBank database are listed in Supplementary Table S1. Amino acid or nucleotide sequences were aligned by the ClustalW method, and phylogenetic trees were constructed using the neighbor-joining method (Saitou and Nei, 1987) in MEGA 7.0 software (Kumar et al., 2016). In the case of genome-based phylogenetic tree, all the compared genome sequences were subjected to whole-genome multiple sequence alignments using the neighbor-joining method (Saitou and Nei, 1987) by the Mauve Multiple Genome Alignments software.^3^ A phylogenetic tree was generated using the aligned genome sequences and visualized by the TreeViewX program^4^. To visualize the putative recombination site in the genome of *M. yongonense* Type I strains, identified ORFs with high sequence similarities to *M. parascrofulaceum* were aligned with other *M. intracellulare* (ATCC 13950^T^, MOTT-02 and MOTT-64), *M. yongonense* Type II (MOTT-36Y and MOTT-H4Y), *M. avium* and *M. parascrofulaceum* strains using the MegAlign program in the DNASTAR package. The possibility of recombination event in the genome of *M. yongonense* Type I strains from *M. parascrofulaceum* was examined using SimPlot program^6^ and boot scanning analysis (Lole et al., 1999). The used parameters are as follows: Window: 200 bp, Step: 20 bp, GapStrip: on, Reps: 100, Kimura (2-parameter), T/t: 2.0, Neighbor-Joining.

### Construction of Recombinant *M. smegmatis* Harboring DNA Mismatch Repair Genes from *M. yongonense* DSM 45126^T^

To generate recombinant *M. smegmatis* harboring DNA mismatch repair genes from *M. yongonense* DSM 45126^T^, approximately 3.8 kb of DNA fragment containing the DNA mismatch repair gene (3,069 bp) and a putative promoter (770 bp) was amplified using a primer set as follows: forward primer; 5′ – TTGCGGCCGCCGACCGAGTTGGCGTGG – 3′ and reverse primer; 5′ – GCTCTAGACCTTTAGACGGCAGTCAG – 3′. The underlined sequence of the forward and reverse primer indicates *Not*I and *Xba*I restriction enzyme sites, respectively. Genomic DNA for *M. yongonense* DSM 45126^T^ was used as a template, and the DNA repair mismatch gene was amplified with *i*-MAX^TM^ II DNA polymerase (iNtRON Biotechnology, Gyeonggi-do, Korea) and a primer set as described above. The PCR amplification condition was as follows: 5 min at 95°C; 40 cycles of 30 s at 95°C, 30 s at 68°C, and 3 min at 72°C; 5 min at 72°C. The PCR amplicon was digested with *Not*I and *Xba*I restriction enzymes and ligated into the pMV306 vector (Blokpoel et al., 2005; Murry et al., 2005; Andreu et al., 2010), which was also digested with the same enzyme.

The pMV306 vector comprising the DNA mismatch repair gene was electroporated into competent *M. smegmatis* mc^2^ 155 using the GenePulser II electroporation apparatus (Bio-Rad, Hercules, CA, United States) (Snapper et al., 1990). Transformants were cultured in Middlebrook 7H9 broth (Difco, Detroit, MI, United States) containing 10% ADC (albumin-dextrose-catalase; Difco) for 3 h and plated onto Middlebrook 7H10 agar plate (supplemented with OADC, oleic acid-albumin-dextrose-catalase; Difco) containing 100 μg/ ml of kanamycin. To check the MutS4 expression in transformants, the mRNA was purified from recombinant *M. smegmatis* carrying MutS4 and empty vector, and RT-PCR was performed using One-step RT-PCR kit (iNtRON Biotechnology, Gyeonggi-do, Korea) with primer sets as follows: forward primer; 5′ – TCC AGG TCC GGC GCA AGG TGT T – 3′ and reverse primer; 5′ – CGC GGG CGG CTG ATG AAG AAG ATA – 3′.

### Examination of the Frequency of Homologous Recombination in Recombinant *M. smegmatis* Harboring DNA Mismatch Repair Gene

A partial RNA polymerase β-subunit gene (*rpoB*) from *M. tuberculosis* was amplified by PCR using genomic DNA from *M. tuberculosis* that had mutations at codon 522 TCG (Ser) → TTG (Leu), designated with ‘317’ related with rifampin resistance (Mariam et al., 2004). The DNA fragment of ‘317’ was provided by the Korean Institute of Tuberculosis (KIT). The partial *rpoB* gene fragments (684 bp) were amplified with a primer set as follows: forward primer, 5′ – CGGGATCCCGTCGGTCGCTATAAGGTCAACA – 3′ and reverse primer, 5′ – CCCAAGCTTCTCGTCGGCGGTCAGGTA – 3′. The underlined sequence of the forward and reverse primers indicates cut sites for *Bam*HI and *Hind*III, respectively. The PCR amplification conditions were as follows: 5 min at 95°C; 30 cycles of 30 s at 95°C, 30 s at 63°C, and 45 s at 72°C; 5 min at 72°C. The amplified fragment was cloned into the *Bam*HI and *Hind*III sites of pSE100 (Guo et al., 2007) to construct pSE100-317.

Constructed pSE100-317 vector was then electroporated into each *M. smegmatis* strain transformed with the present DNA mismatch repair gene or an empty vector pMV306, as described above. The transformants were then plated onto 7H10 agar plates with 50 μg/ml of hygromycin and incubated for 72 h at 37°C. After that, colonies were picked and suspended in 7H9 broth with 50 μg/ml of hygromycin and cultured for 72 h at 37°C. The cultured bacterial sample was adjusted to 0.2 OD (optical density at 600 nm) and plated onto the 7H10 agar plate with 100 μg/ml of rifampin. Colonies grown on the rifampin 7H10 agar plates were judged as potential recombinants. The number of colonies was counted after 3 days incubation and the *rpoB* gene was amplified by PCR using the primers 7940F (forward, 5′ – TCAAGGAGAAGCGCTACGACC – 3′) and MR (reverse, 5′ – TCGATCGGGCACATCCGG – 3′) from the randomly selected colonies. PCR amplicons were then sequenced using the 7940F and MR primers. Recombination-emerging colonies were identified by having *M. tuberculosis*-specific SNPs, especially rifampin resistant related SNPs in their *rpoB* sequences. Also, the lengths of the recombined *rpoB* gene was calculated by determination the boundary between the *M. smegmatis*- and *M. tuberculosis*-specific SNP.

## Author Contributions

By-JK and B-RK performed the experiments and sequence analyses. Bu-JK and Y-HK designed and interpreted the experiments. Bu-JK wrote the manuscript.

## Conflict of Interest Statement

The authors declare that the research was conducted in the absence of any commercial or financial relationships that could be construed as a potential conflict of interest.

## Footnotes

1 http://www.pathogenomics.sfu.ca/islandviewer/browse/

2 http://www.pathogenomics.sfu.ca/islandviewer/browse/

3 http://darlinglab.org/mauve/mauve.html

4 http://darwin.zoology.gla.ac.uk/~rpage/treeviewx/

## References

[B1] Achtman M. Wagner M. (2008). Microbial diversity and the genetic nature of microbial species. *Nat. Rev. Microbiol.* 6 431–440. 10.1038/nrmicro1872 18461076

[B2] Andreu N. Zelmer A. Fletcher T. Elkington P. T. Ward T. H. Ripoll J. (2010). Optimisation of bioluminescent reporters for use with Mycobacteria. *PLOS ONE* 5:e10777. 10.1371/journal.pone.0010777 20520722 PMC2875389

[B3] Arruda S. Bomfim G. Knights R. Huima-Byron T. Riley L. W. (1993). Cloning of an *M. tuberculosis* DNA fragment associated with entry and survival inside cells. *Science* 261 1454–1457. 10.1126/science.8367727 8367727

[B4] Banasik M. Sachadyn P. (2014). Conserved motifs of MutL proteins. *Mutat. Res.* 769 69–79. 10.1016/j.mrfmmm.2014.07.006 25771726

[B5] Bertelli C. Laird M. R. Williams K. P. Lau B. Y. Hoad G. Winsor G. L. (2017). IslandViewer 4: expanded prediction of genomic islands for larger-scale datasets. *Nucleic Acids Res.* 10.1093/nar/gkx343 [Epub ahead of print]. 28472413 PMC5570257

[B6] Blokpoel M. C. Murphy H. N. O’Toole R. Wiles S. Runn E. S. Stewart G. R. (2005). Tetracycline-inducible gene regulation in mycobacteria. *Nucleic Acids Res.* 33:e22. 10.1093/nar/gni023 15687380 PMC548381

[B7] Castaneda-Garcia A. Prieto A. I. Rodriguez-Beltran J. Alonso N. Cantillon D. Costas C. (2017). A non-canonical mismatch repair pathway in prokaryotes. *Nat. Commun.* 8:14246. 10.1038/ncomms14246 28128207 PMC5290159

[B8] Cole S. T. Brosch R. Parkhill J. Garnier T. Churcher C. Harris D. (1998). Deciphering the biology of *Mycobacterium tuberculosis* from the complete genome sequence. *Nature* 393 537–544. 10.1038/31159 9634230

[B9] Cole S. T. Eiglmeier K. Parkhill J. James K. D. Thomson N. R. Wheeler P. R. (2001). Massive gene decay in the leprosy bacillus. *Nature* 409 1007–1011. 10.1038/35059006 11234002

[B10] Didelot X. Maiden M. C. (2010). Impact of recombination on bacterial evolution. *Trends Microbiol.* 18 315–322. 10.1016/j.tim.2010.04.002 20452218 PMC3985120

[B11] Didelot X. Meric G. Falush D. Darling A. E. (2012). Impact of homologous and non-homologous recombination in the genomic evolution of *Escherichia coli*. *BMC Genomics* 13:256. 10.1186/1471-2164-13-256 22712577 PMC3505186

[B12] Forrellad M. A. Klepp L. I. Gioffre A. Garcia J. S. Y. Morbidoni H. R. Santangelo M. D. (2013). Virulence factors of the *Mycobacterium tuberculosis* complex. *Virulence* 4 3–66. 10.4161/viru.22329 23076359 PMC3544749

[B13] Fraser C. Alm E. J. Polz M. F. Spratt B. G. Hanage W. P. (2009). The bacterial species challenge: making sense of genetic and ecological diversity. *Science* 323 741–746. 10.1126/science.1159388 19197054

[B14] Guo X. V. Monteleone M. Klotzsche M. Kamionka A. Hillen W. Braunstein M. (2007). Silencing essential protein secretion in *Mycobacterium smegmatis* by using tetracycline repressors. *J. Bacteriol.* 189 4614–4623. 10.1128/Jb.00216-07 17483222 PMC1913471

[B15] Harfe B. D. Jinks-Robertson S. (2000). DNA mismatch repair and genetic instability. *Annu. Rev. Genet.* 34 359–399. 10.1146/annurev.genet.34.1.359 11092832

[B16] Hsiao W. Wan I. Jones S. J. Brinkman F. S. L. (2003). IslandPath: aiding detection of genomic islands in prokaryotes. *Bioinformatics* 19 418–420. 10.1093/bioinformatics/btg004 12584130

[B17] Iyer R. R. Pluciennik A. Burdett V. Modrich P. L. (2006). DNA mismatch repair: functions and mechanisms. *Chem. Rev.* 106 302–323. 10.1021/cr0404794 16464007

[B18] Kim B. J. Choi B. S. Choi I. Y. Lee J. H. Chun J. Hong S. H. (2012a). Complete genome sequence of *Mycobacterium intracellulare* clinical strain MOTT-36Y, belonging to the INT5 genotype. *J. Bacteriol.* 194 4141–4142. 10.1128/JB.00752-12 22815454 PMC3416561

[B19] Kim B. J. Choi B. S. Lim J. S. Choi I. Y. Kook Y. H. (2012b). Complete genome sequence of *Mycobacterium intracellulare* clinical strain MOTT-64, belonging to the INT1 genotype. *J. Bacteriol.* 194:3268. 10.1128/JB.00471-12 22628501 PMC3370864

[B20] Kim B. J. Choi B. S. Lim J. S. Choi I. Y. Lee J. H. Chun J. (2012c). Complete genome sequence of *Mycobacterium intracellulare* strain ATCC 13950(T). *J. Bacteriol.* 194:2750. 10.1128/JB.00295-12 22535933 PMC3347195

[B21] Kim B. J. Choi B. S. Lim J. S. Choi I. Y. Lee J. H. Chun J. (2012d). Complete genome sequence of *Mycobacterium intracellulare* clinical strain MOTT-02. *J. Bacteriol.* 194:2771. 10.1128/JB.00365-12 22535946 PMC3347180

[B22] Kim B. J. Hong S. H. Kook Y. H. (2013a). Molecular evidence of lateral gene transfer in rpoB gene of *Mycobacterium yongonense* strains via multilocus sequence analysis. *PLOS ONE* 8:e51846. 10.1371/journal.pone.0051846 23382812 PMC3561371

[B23] Kim B. J. Kim B. R. Lee S. Y. Seok S. H. Kook Y. H. (2013b). Whole-genome sequence of a novel species, *Mycobacterium yongonense* DSM 45126T. *Genome Announc.* 1:e00604-13. 10.1128/genomeA.00604-13 23929490 PMC3738906

[B24] Kim B. J. Math R. K. Jeon C. O. Yu H. K. Park Y. G. Kook Y. H. (2013c). *Mycobacterium yongonense* sp. nov., a slow-growing non-chromogenic species closely related to *Mycobacterium intracellulare*. *Int. J. Syst. Evol. Microbiol.* 63(Pt 1), 192–199. 10.1099/ijs.0.037465-0 22427442

[B25] Kim B. J. Kim B. R. Lee S. Y. Kim G. N. Kook Y. H. (2016). Molecular taxonomic evidence for two distinct genotypes of *Mycobacterium yongonense* via genome-based phylogenetic analysis. *PLOS ONE* 11:e0152703. 10.1371/journal.pone.0152703 27031100 PMC4816341

[B26] Kim B. J. Kim G. N. Kim B. R. Shim T. S. Kook Y. H. Kim B. J. (2017). Phylogenetic analysis of *Mycobacterium massiliense* strains having recombinant *rpoB* gene laterally transferred from *Mycobacterium abscessus*. *PLOS ONE* 12:e0179237. 10.1371/journal.pone.0179237 28604829 PMC5467896

[B27] Kim B. J. Kim K. Kim B. R. Kook Y. H. (2015). Identification of IS*Myo*2, a novel insertion sequence element of IS21 family and its diagnostic potential for detection of *Mycobacterium yongonense*. *BMC Genomics* 16:794. 10.1186/s12864-015-1978-2 26472562 PMC4608216

[B28] Kumar A. Bose M. Brahmachari V. (2003). Analysis of expression profile of mammalian cell entry (mce) operons of *Mycobacterium tuberculosis*. *Infect. Immun.* 71 6083–6087. 10.1128/IAI.71.10.6083-6087.2003 14500535 PMC201107

[B29] Kumar S. Stecher G. Tamura K. (2016). MEGA7: molecular evolutionary genetics analysis version 7.0 for bigger datasets. *Mol. Biol. Evol.* 33 1870–1874. 10.1093/molbev/msw054 27004904 PMC8210823

[B30] Langille M. G. I. Brinkman F. S. L. (2009). IslandViewer: an integrated interface for computational identification and visualization of genomic islands. *Bioinformatics* 25 664–665. 10.1093/bioinformatics/btp030 19151094 PMC2647836

[B31] Langille M. G. I. Hsiao W. W. L. Brinkman F. S. L. (2008). Evaluation of genomic island predictors using a comparative genomics approach. *BMC Bioinformatics* 9:329. 10.1186/1471-2105-9-329 18680607 PMC2518932

[B32] Lee H. Kim B. J. Kim K. Hong S. H. Kook Y. H. (2013). Whole-genome sequence of *Mycobacterium intracellulare* clinical strain MOTT-H4Y, belonging to INT5 genotype. *Genome Announc.* 1:e00006-13. 10.1128/genomeA.00006-13 23472222 PMC3587921

[B33] Lin Z. Nei M. Ma H. (2007). The origins and early evolution of DNA mismatch repair genes–multiple horizontal gene transfers and co-evolution. *Nucleic Acids Res.* 35 7591–7603. 10.1093/nar/gkm921 17965091 PMC2190696

[B34] Lole K. S. Bollinger R. C. Paranjape R. S. Gadkari D. Kulkarni S. S. Novak N. G. (1999). Full-length human immunodeficiency virus type 1 genomes from subtype C-infected seroconverters in India, with evidence of intersubtype recombination. *J. Virol.* 73 152–160. 9847317 10.1128/jvi.73.1.152-160.1999 PMC103818

[B35] Macheras E. Roux A. L. Bastian S. Leao S. C. Palaci M. Sivadon-Tardy V. (2011). Multilocus sequence analysis and *rpoB* sequencing of *Mycobacterium abscessus* (sensu lato) strains. *J. Clin. Microbiol.* 49 491–499. 10.1128/JCM.01274-10 21106786 PMC3043527

[B36] Mariam D. H. Mengistu Y. Hoffner S. E. Andersson D. I. (2004). Effect of *rpoB* mutations conferring rifampin resistance on fitness of *Mycobacterium tuberculosis*. *Antimicrob. Agents Chemother.* 48 1289–1294. 10.1128/AAC.48.4.1289-1294.2004 15047531 PMC375340

[B37] Miller J. L. Velmurugan K. Cowan M. J. Briken V. (2010). The type I NADH dehydrogenase of *Mycobacterium tuberculosis* counters phagosomal NOX2 activity to inhibit TNF-alpha-mediated host cell apoptosis. *PLOS Pathog.* 6:e1000864. 10.1371/journal.ppat.1000864 20421951 PMC2858756

[B38] Mizrahi V. Andersen S. J. (1998). DNA repair in *Mycobacterium tuberculosis*. What have we learnt from the genome sequence? *Mol. Microbiol.* 29 1331–1339. 10.1046/j.1365-2958.1998.01038.x 9781872

[B39] Modrich P. Lahue R. (1996). Mismatch repair in replication fidelity, genetic recombination, and cancer biology. *Annu. Rev. Biochem.* 65 101–133. 10.1146/annurev.bi.65.070196.000533 8811176

[B40] Murry J. Sassetti C. M. Moreira J. Lane J. Rubin E. J. (2005). A new site-specific integration system for mycobacteria. *Tuberculosis* 85 317–323. 10.1016/j.tube.2005.08.016 16256438

[B41] Ochman H. Lawrence J. G. Groisman E. A. (2000). Lateral gene transfer and the nature of bacterial innovation. *Nature* 405 299–304. 10.1038/35012500 10830951

[B42] Ogata H. Ray J. Toyoda K. Sandaa R. A. Nagasaki K. Bratbak G. (2011). Two new subfamilies of DNA mismatch repair proteins (MutS) specifically abundant in the marine environment. *ISME J.* 5 1143–1151. 10.1038/ismej.2010.210 21248859 PMC3146287

[B43] Reenan R. A. Kolodner R. D. (1992). Characterization of insertion mutations in the *Saccharomyces cerevisiae* MSH1 and MSH2 genes: evidence for separate mitochondrial and nuclear functions. *Genetics* 132 975–985. 1334021 10.1093/genetics/132.4.975 PMC1205253

[B44] Sachadyn P. (2010). Conservation and diversity of MutS proteins. *Mutat. Res.* 694 20–30. 10.1016/j.mrfmmm.2010.08.009 20833188

[B45] Saitou N. Nei M. (1987). The neighbor-joining method: a new method for reconstructing phylogenetic trees. *Mol. Biol. Evol.* 4 406–425. 3447015 10.1093/oxfordjournals.molbev.a040454

[B46] Sapriel G. Konjek J. Orgeur M. Bouri L. Frezal L. Roux A. L. (2016). Genome-wide mosaicism within *Mycobacterium abscessus*: evolutionary and epidemiological implications. *BMC Genomics* 17:118. 10.1186/s12864-016-2448-1 26884275 PMC4756508

[B47] Sheppard S. K. McCarthy N. D. Falush D. Maiden M. C. (2008). Convergence of *Campylobacter* species: implications for bacterial evolution. *Science* 320 237–239. 10.1126/science.1155532 18403712

[B48] Snapper S. B. Melton R. E. Mustafa S. Kieser T. Jacobs W. R. Jr. (1990). Isolation and characterization of efficient plasmid transformation mutants of *Mycobacterium smegmatis*. *Mol. Microbiol.* 4 1911–1919. 10.1111/j.1365-2958.1990.tb02040.x 2082148

[B49] Surtees J. A. Argueso J. L. Alani E. (2004). Mismatch repair proteins: key regulators of genetic recombination. *Cytogenet. Genome Res.* 107 146–159. 10.1159/000080593 15467360

[B50] Umar A. Kunkel T. A. (1996). DNA-replication fidelity, mismatch repair and genome instability in cancer cells. *Eur. J. Biochem.* 238 297–307. 10.1111/j.1432-1033.1996.0297z.x 8681938

[B51] Velmurugan K. Chen B. Miller J. L. Azogue S. Gurses S. Hsu T. (2007). *Mycobacterium tuberculosis nuoG* is a virulence gene that inhibits apoptosis of infected host cells. *PLOS Pathog.* 3:e110. 10.1371/journal.ppat.0030110 17658950 PMC1924871

[B52] Vulic M. Dionisio F. Taddei F. Radman M. (1997). Molecular keys to speciation: DNA polymorphism and the control of genetic exchange in enterobacteria. *Proc. Natl. Acad. Sci. U.S.A.* 94 9763–9767. 10.1073/pnas.94.18.9763 9275198 PMC23264

[B53] Zhang F. Xie J. P. (2011). Mammalian cell entry gene family of *Mycobacterium tuberculosis*. *Mol. Cell. Biochem.* 352 1–10. 10.1007/s11010-011-0733-5 21258845

