# Supplementary material for: Role of the DNA Mismatch Repair Gene MutS4 in Driving the Evolution of Mycobacterium yongonense Type I via Homologous Recombination
Source: Front Microbiol. 2017 Dec 20;8:2578. doi: 10.3389/fmicb.2017.02578 (PMC5742357; doi:10.3389/fmicb.2017.02578)
Supplement: Supplementary file 1 [file Data_Sheet_1.pdf]

Supplementary Figures and Tables

Role of the DNA mismatch repair gene *MutS4* in driving the evolution of *Mycobacterium yongonense* Type I via homologous recombination

Byoung-Jun Kim, Bo-Ram Kim, Yoon-Hoh Kook and Bum-Joon Kim*

Department of Microbiology and Immunology, Biomedical Sciences, Liver Research Institute and Cancer Research Institute, College of Medicine, Seoul National University, Seoul, Korea

***Author for correspondence:** Bum-Joon Kim, phD,

E-mail : kbumjoon@snu.ac.kr.

(A) OEM_44200 (sim14)

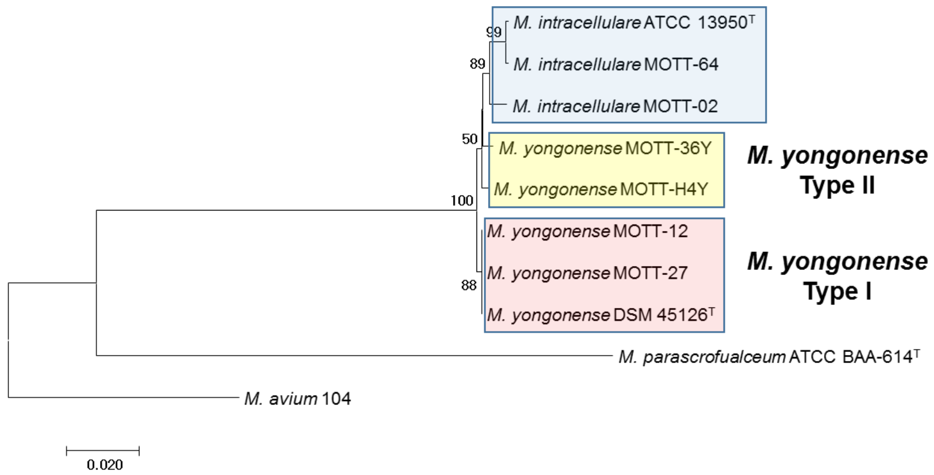

(B) OEM_44190 (ABC transporter)

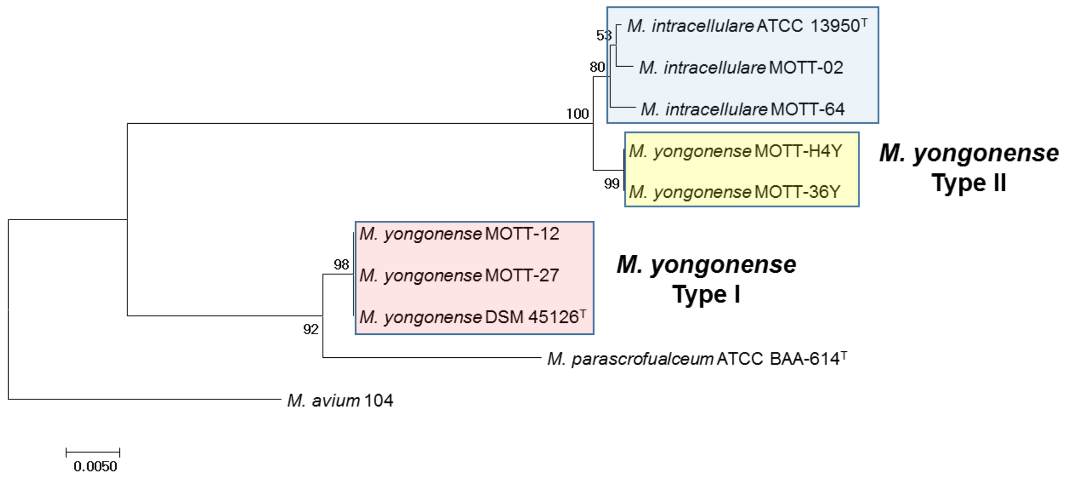

(C) OEM_44170 (*rpoC*)

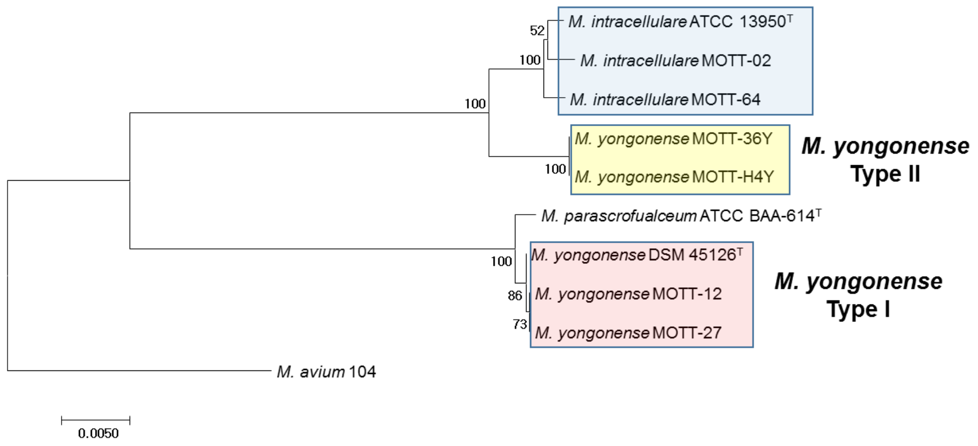

(D) OEM_44160 (endonuclease IV)

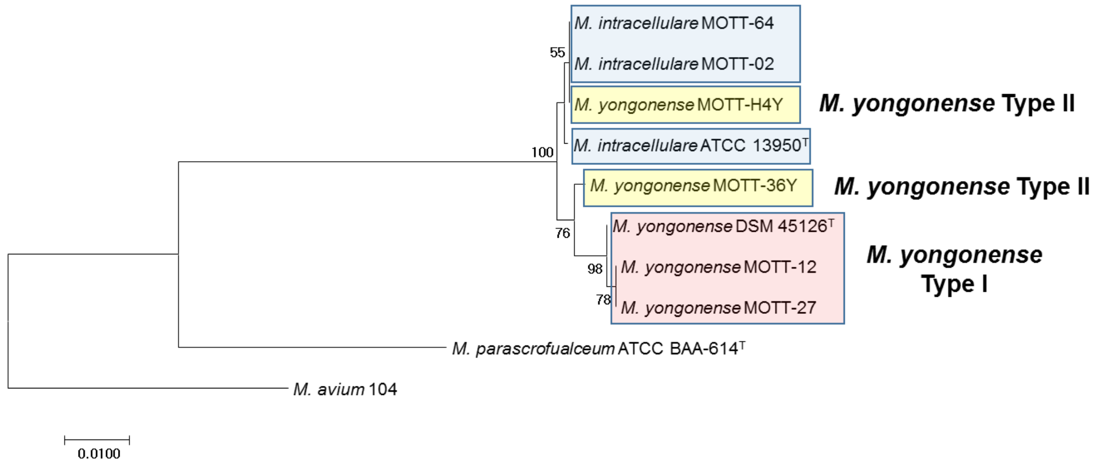

Supplementary Figure S1. Phylogenetic analysis based on ORFs on the border within first putative lateral gene transferred region (OEM_44170~44190). (A and D) Trees based on adjacent ORFs (OEM_44200; sim14 and OEM_44160; endonuclease IV) from putative lateral gene transferred region. (B and C) Trees based on utative lateral transferred ORFs (OEM_44190; ABC transporter and OEM_44170; *rpoC*) in *M. yongonense* Type I strains from *M. parascrofulaceum.* Red square, *M. yongonense* Type II strains; yellow square, *M. yongonense* Type I strains; blue square, *M. intracellulare* strains. Bootstrap values were calculated from 1,000 replications and values <50% were not shown. Bar indicates numbers of base substitutions per site. ORFs from *M. avium* 104 were used as an out-group.

(A) OEM_08020 (hypothetical protein)

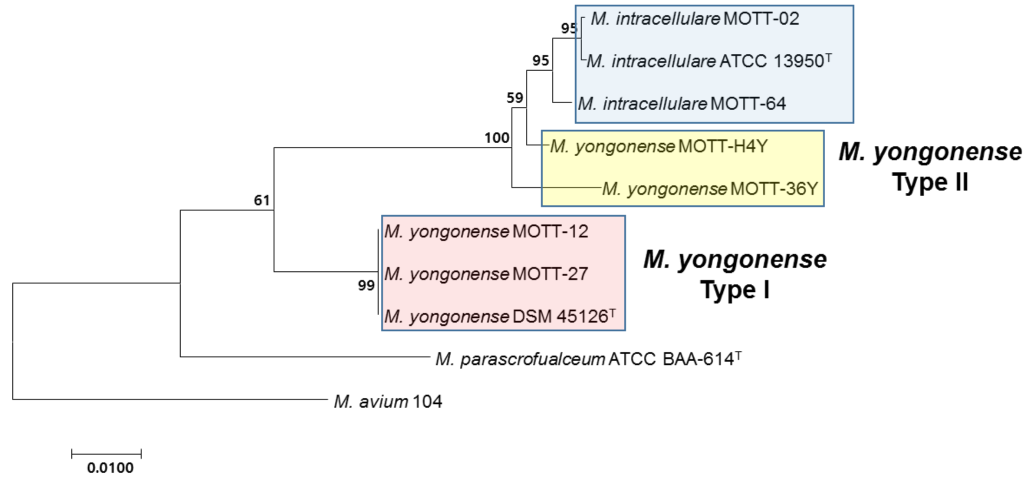

(B) OEM_08030 [Rieske (2Fe-2S) domain protein]

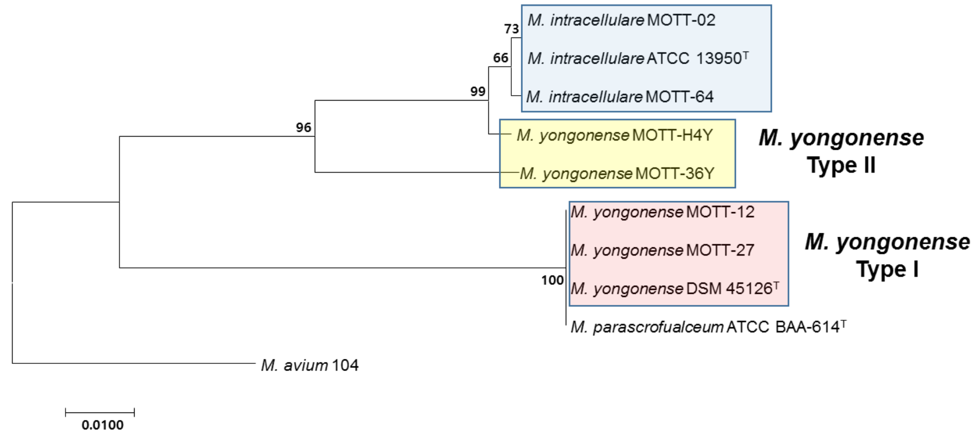

(C) OEM_08590 (fatty oxidation complex)

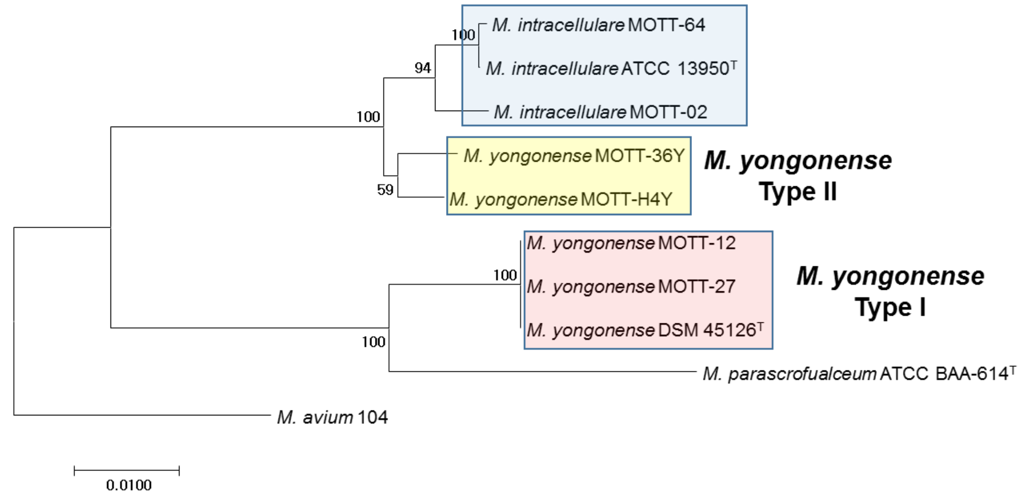

(D) OEM_08600 (putative acyl-CoA dehydrogenase)

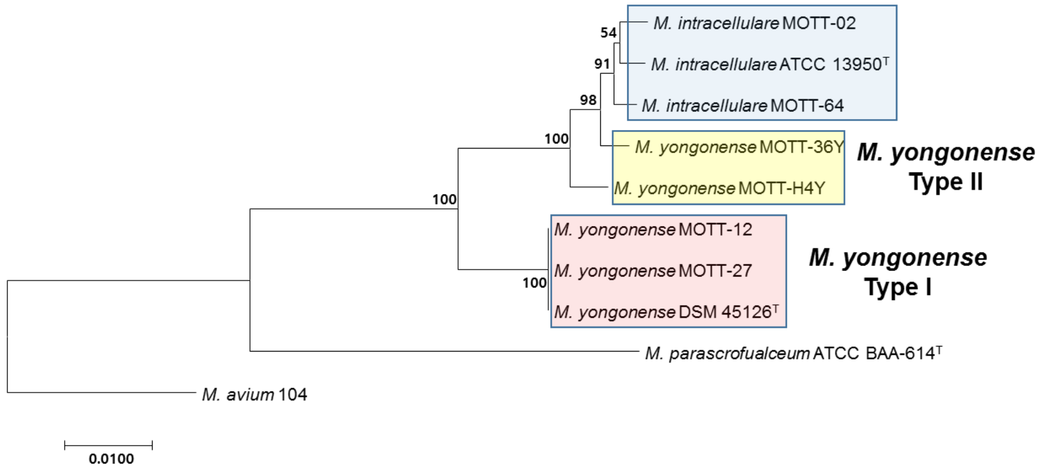

Supplementary Figure S2. Phylogenetic analysis based on ORFs on the border within the second putative lateral gene transferred region (OEM_08030~08590). (A and D) Trees based on adjacent ORFs (OEM_08020; hypothetical protein and OEM_08600; putative acyl-CoA dehydrogenase) from putative lateral gene transferred region. (B and C) Trees based on putative lateral transferred ORFs [OEM_08030; Rieske (2Fe-2S) domain protein and OEM_08590; fatty oxidation complex) in *M. yongonense* Type I strains from *M. parascrofulaceum.* Red square, *M. yongonense* Type II strains; yellow square, *M. yongonense* Type I strains; blue square, *M. intracellulare* strains. Bootstrap values were calculated from 1,000 replications and values <50% were not shown. Bar indicates numbers of base substitutions per site. ORFs from *M. avium* 104 were used as an out-group.

(A) OEM_44190 (ABC transporter)

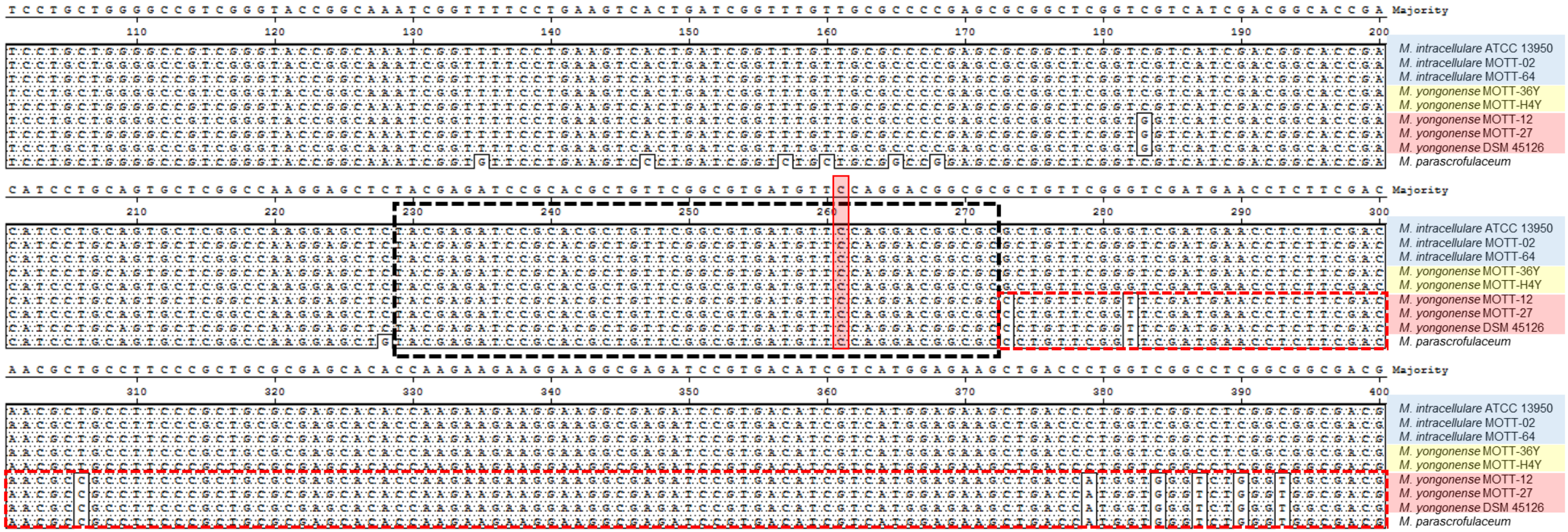

(B) OEM_44170 (*rpoC*)

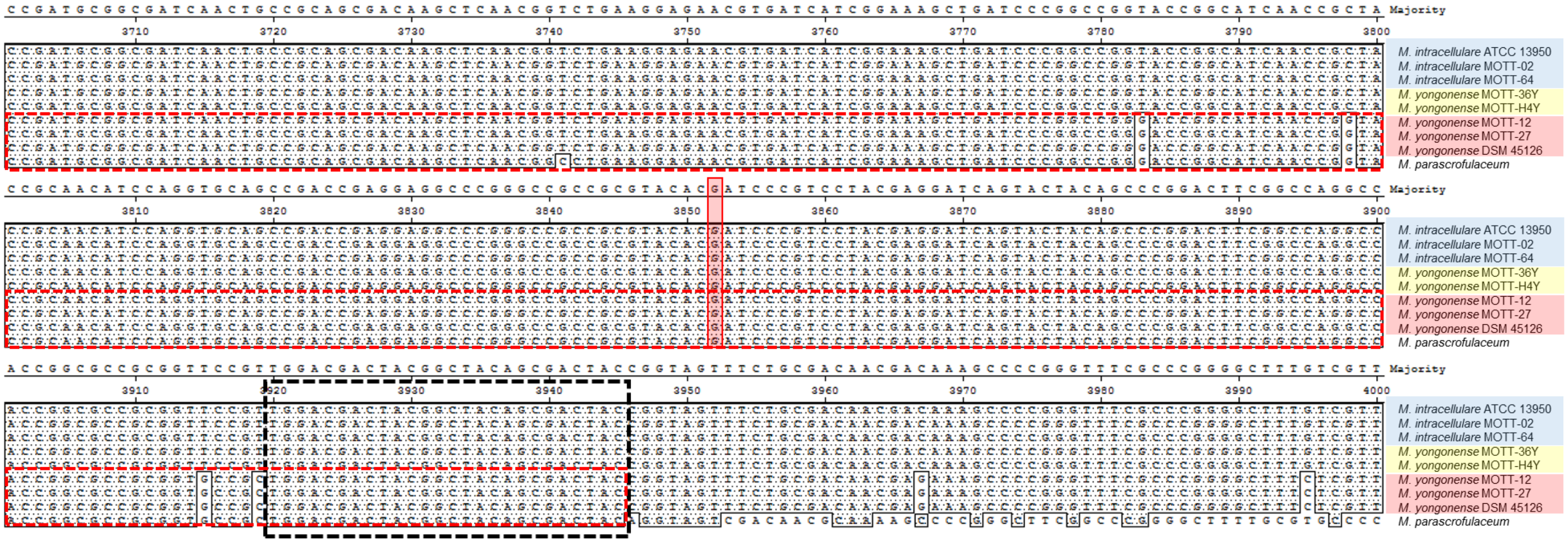

(C) OEM_08020

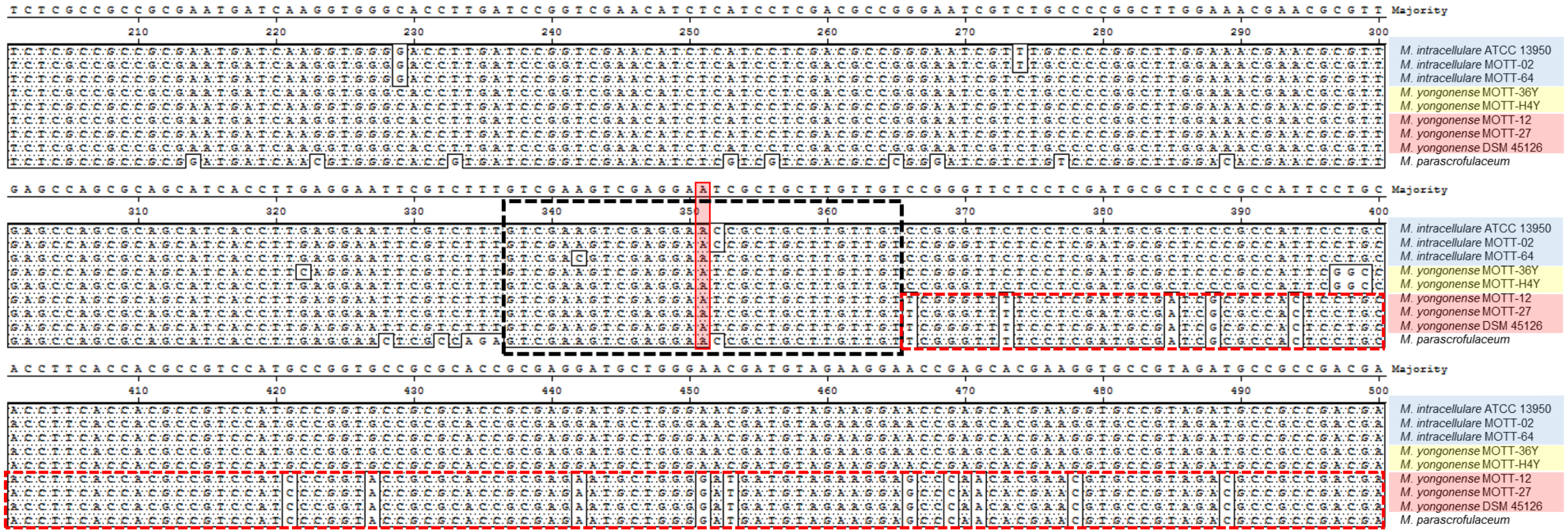

(D) OEM_08600

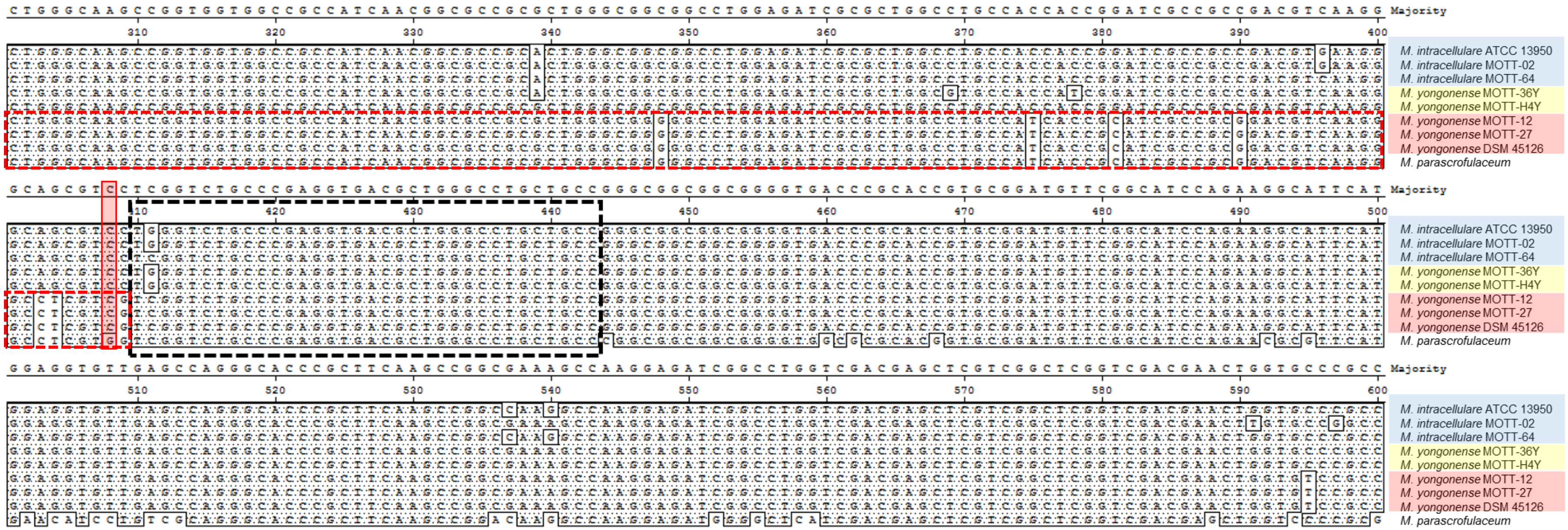

Supplementary Figure S3. Multiple alignments of sequences in a putative recombination site from *M. intracellulare* (ATCC 13950^{T}, MOTT-02 and MOTT-64), *M. yongonense* Type II (MOTT-36Y and MOTT-H4Y) and *M. parascrofulaceum* strains to *M. yongonense* Type I (DSM 45126^{T}, MOTT-12 and MOTT-27) strains. (A and B) First recombination site (from OEM_44170 to 44190). (C and D) Second recombination site (from OEM_08030 to 08590). Each figure indicate multiple alignments of sequences corresponding to each putative LGT region. Red spaced box indicates breakpoints determined by BootStrap analysis. Black dashed box indicates putative recombinated region. Red dashed box indicates the identical sequence region between *M. yongonense* Type I and *M. parascrofulaceum* strains.

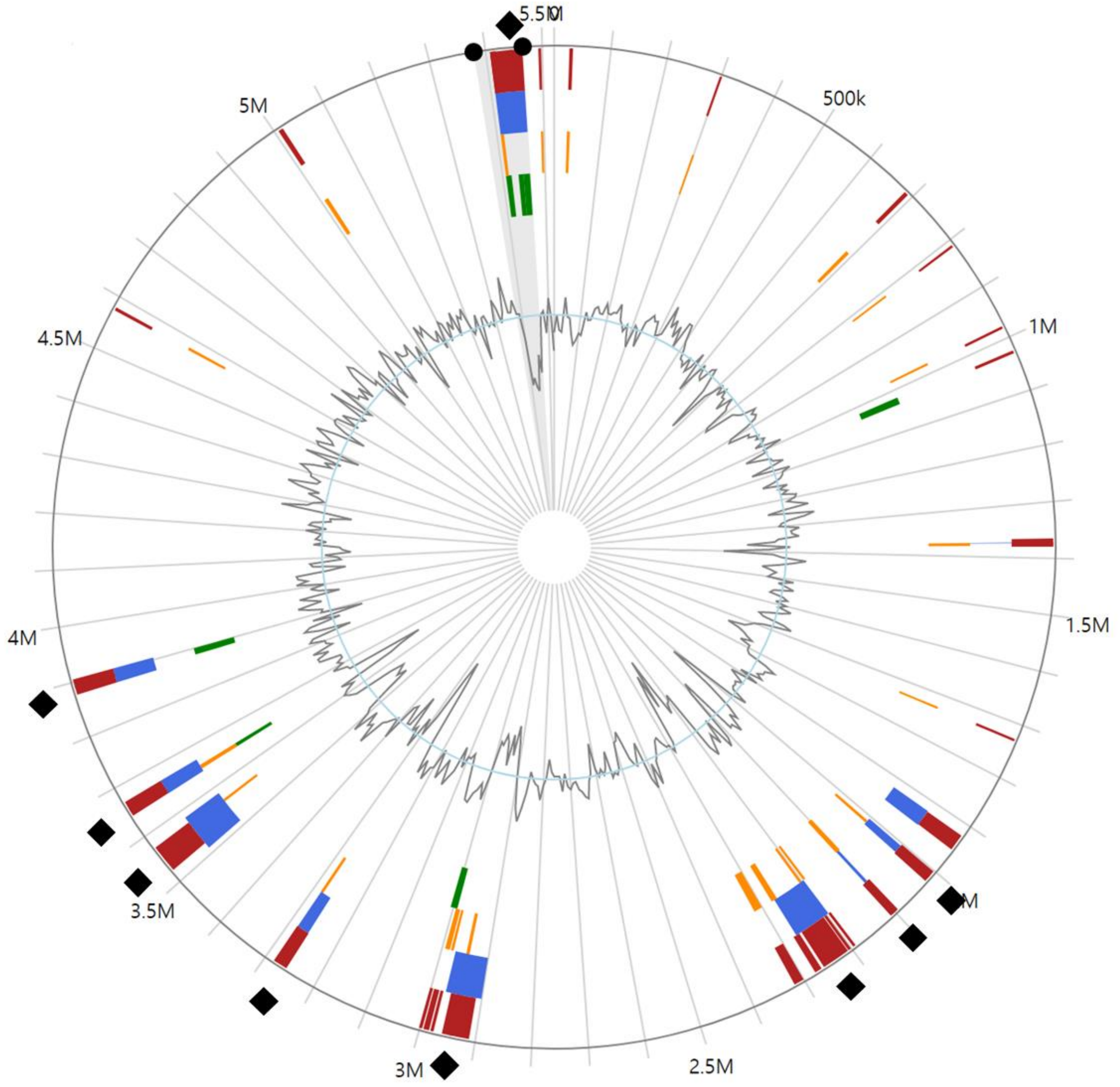

Supplementary Figure S4. Visualization of genomic islands in *M. yongonense* DSM 45126^{T} genome using IslandViewer 4 program. Each putative genomic island is labelled with color depending on island prediction methods: red, integrated; green, IslandPick; orange, SIGI-HMM; blue, IslandPath-DIMOB. Inner black line plot represents G+C content (%) of genome. Loci predicted by three or four prediction methods were selected (black centered diamond) and grey spaced locus includes DNA mismatch repair genes, *MutS4A* and *MutS4B*.

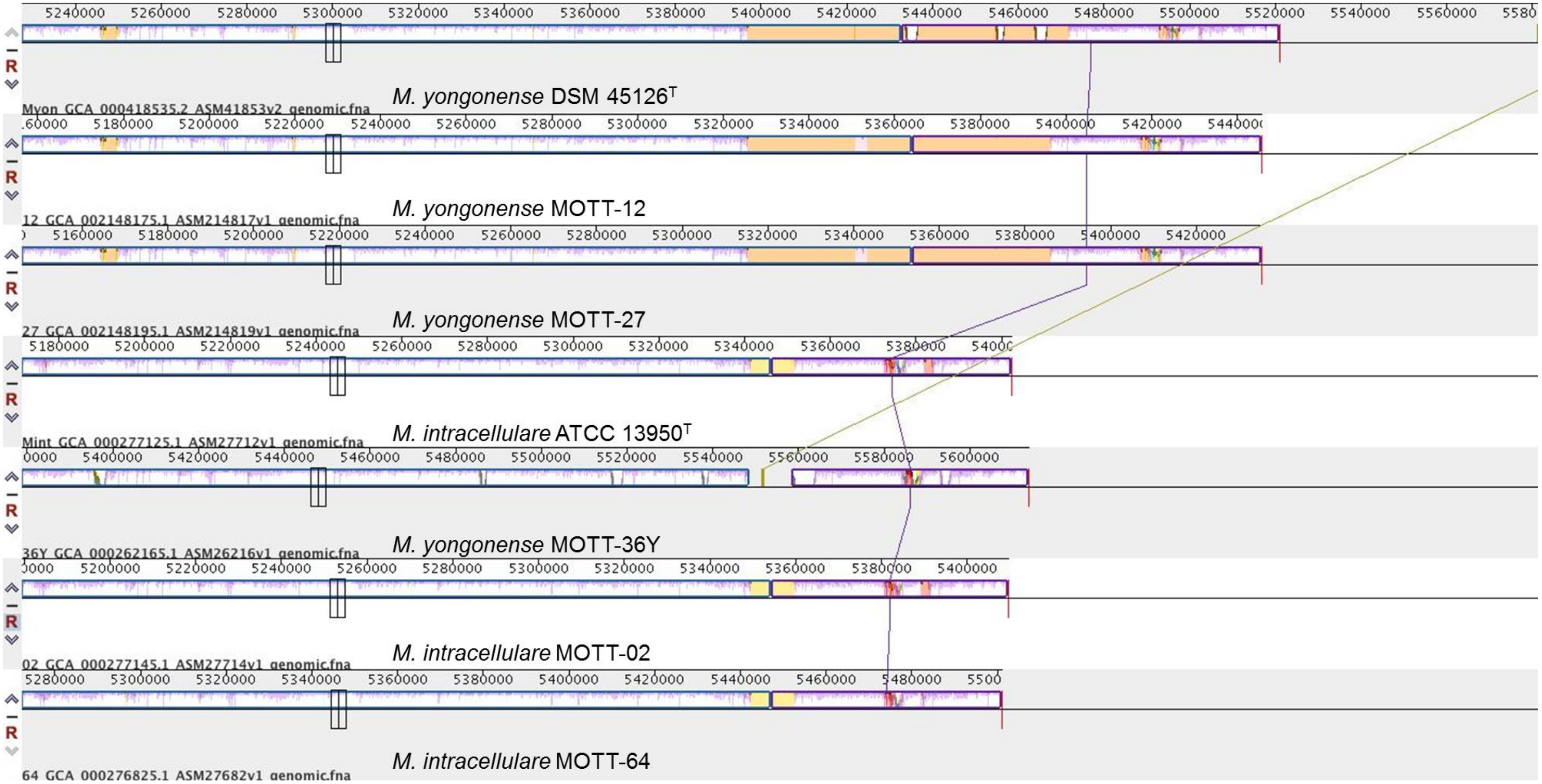

Supplementary Figure S5. Genome sequences of *M. yongonense* Type I (DSM 45126^T, MOTT-12 and MOTT-27), *M. intracellulare* (ATCC 13950^T, MOTT-02 and MOTT-64) and *M. yongonense* Type II (MOTT-36Y), especially the locus includes DNA mismatch repair genes (colored with peach in the genomes of *M. yongonense* Type I strains) were used for multiple genome alignment which was performed using Mauve software (http://darlinglab.org/mauve/mauve.html).

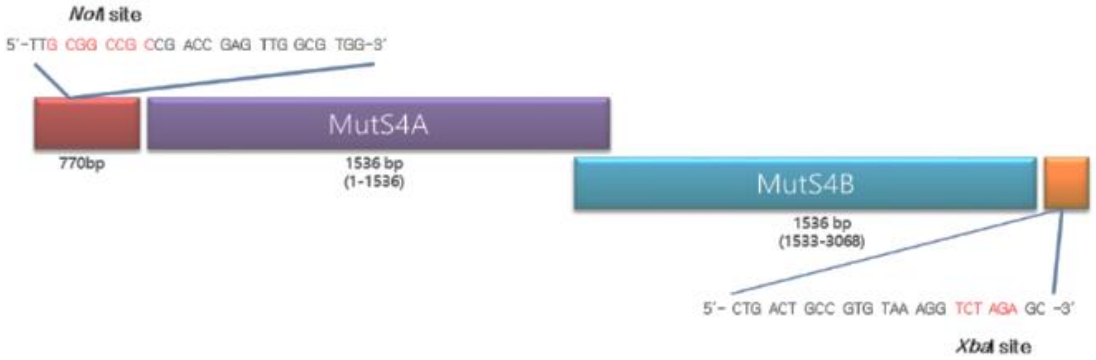

Supplementary Figure S6. Schematic representation of DNA mismatch repair genes from *M. yongonense* DSM 45126^{T}. Primer sequences are indicated in forward and reverse regions.

(A)

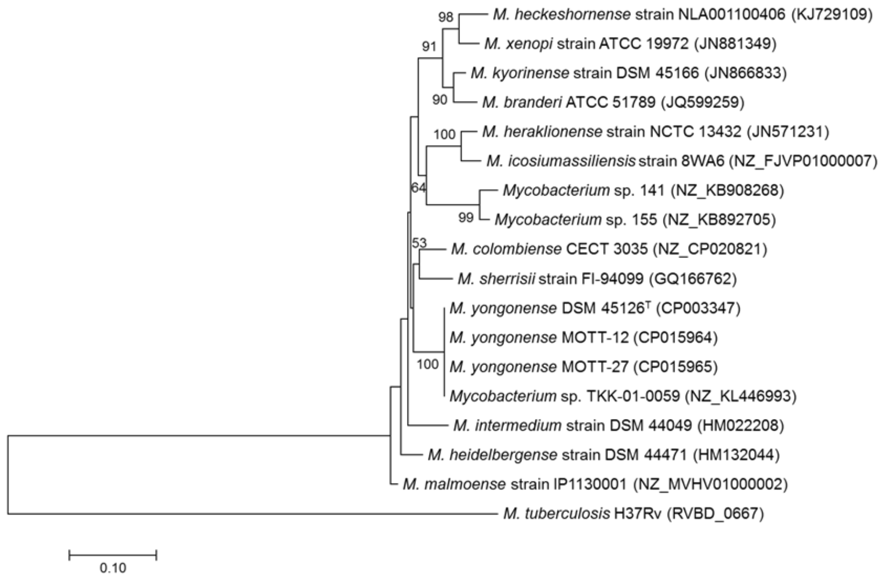

(B)

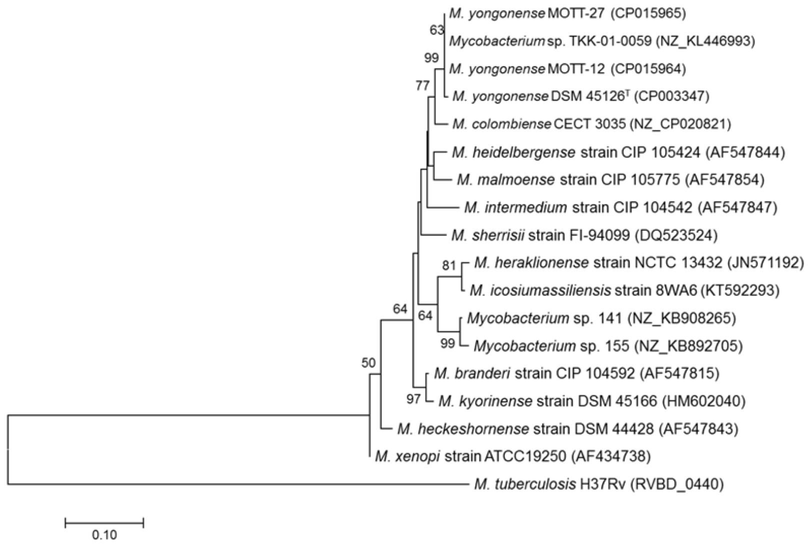

Supplementary Figure S7. Neighbor-joining trees based on partial (A) *rpoB* (711 bp) and (B) *hsp65* (~400 bp) gene sequences of *Mycobacterium* strains with MutS4 homologs in their genome. All nucleotide sequences were retrieved from the GenBank database, and accession numbers are indicated in brackets. Bootstrap values were calculated from 1,000 replications and those below 50 % re not shown. Sequences of *M. tuberculosis* H37Rv were used as an out-group in each tree. Scale bar indicates numbers of substitutions per nucleotide position.

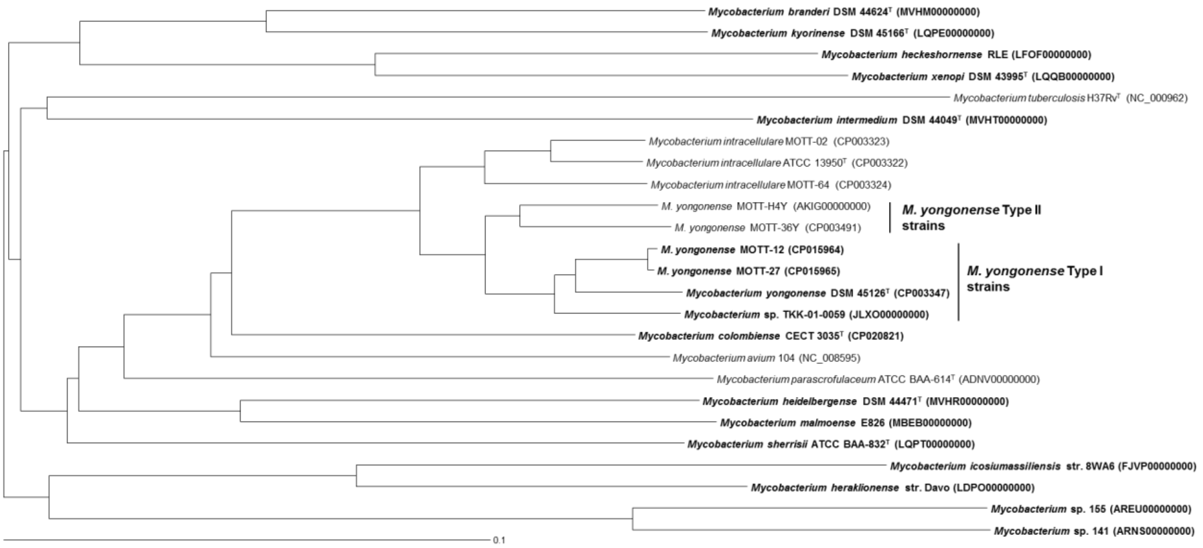

Supplementary Figure S8. Phylogenetic tree based on whole-genome sequences of *Mycobacterium* strains with MutS4 homologs in their genome (marked in bold). The tree was generated using the neighbor-joining method by the Mauve Genome Alignment software and visualized by the TreeViewX program. The bar indicates the number of substitutions per nucleotide position.

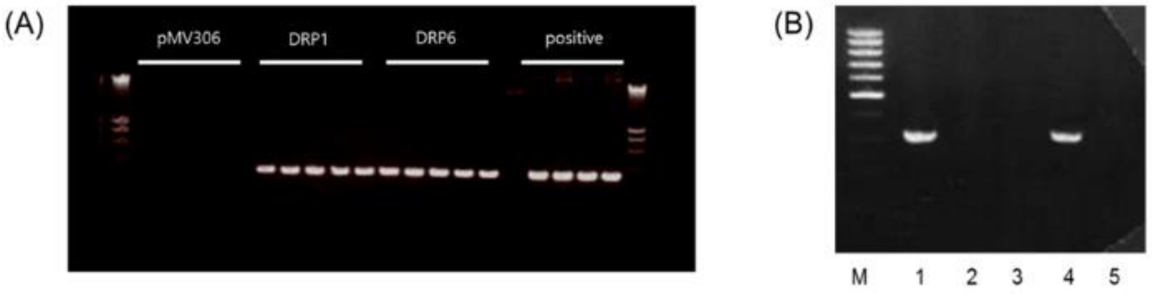

Supplementary Figure S9. Confirmation of recombinant *M. smegmatis* harboring DNA mismatch repair gene by colony PCR and RT-PCR. (A) Colony PCR results. pMV306, recombinant *M. smegmatis* harboring an empty vector, pMV306; DR1 and DR6, recombinant *M. smegmatis* harboring DNA mismatch repair gene; positive control, pMV306 vector cloned with DNA mismatch repair gene. (B) RT-PCR results. M, 100 bp DNA ladder; 1, *M. yongonense* DSM 45126^T; 2, *M. intracellulare* ATCC 13950^T; 3, recombinant *M. smegmatis* harboring an empty vector, pMV306; 4, recombinant *M. smegmatis* harboring DNA mismatch repair gene; 5, Negative control.

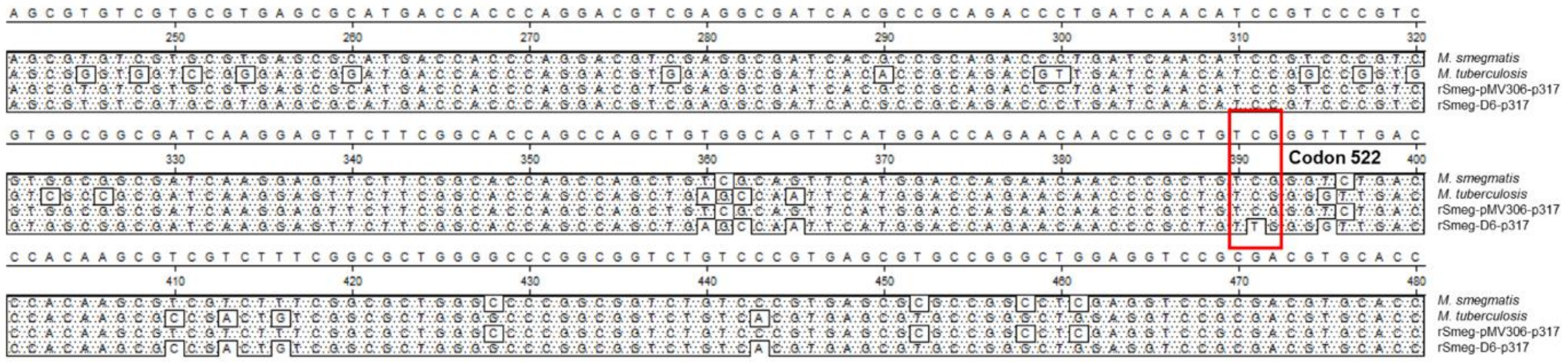

Supplementary Figure S10. Representative multiple alignment of sequences in putative homologous recombination sites from each transformed recombinant *M. smegmatis*. As a control, *rpoB* sequences of *M. smegmatis* and *M. tuberculosis* were also aligned. Red box indicates mutation codons that confer resistance to rifampin in *M. tuberculosis*. rSmeg-pMV306-p317, recombinant *M. smegmatis* harboring empty vector and transformed with pSE100-317 *rpoB*; rSmeg-D6-p317, recombinant *M. smegmatis* harboring DNA mismatch repair gene and transformed with pSE100-317 *rpoB.*

Supplementary Table S1. Sequence similarities between ORFs which consist of the putative transferred regions in *M. yongonense* DSM 45126[T] and other *Mycobacterium* species

(A) TR1, [*rpoBC* operon (OEM_44170~44190)]

ORFs of Myon[a]	Gene annotation	Sequence similarities (%)								
		Mint[b]	MOTT-02	MOTT-64	MOTT-36Y	MOTT-H4Y	MOTT-12	MOTT-27	Mpara[c]	Mav[d]
OEM_44170	DNA-directed RNA polymerase subunit beta prime	94	94	94	94	94	99	99	99	95
OEM_44180	DNA-directed RNA polymerase subunit beta	94	94	94	94	94	99	99	99	94
OEM_44190	Sulfate/thiosulfate ABC superfamily ATP binding cassette transporter, ABC protein	95	95	94	94	94	100	100	97	93

(B) TR2, (OEM_08030~08590)

ORFs of Myon[a]	Gene annotation	Sequence similarities (%)								
		Mint[b]	MOTT-	MOTT-	MOTT-	MOTT-	MOTT-	MOTT-	Mpara[c]	Mav[d]

			02	64	36Y	H4Y	12	27		
OEM_08030	rieske (2Fe-2S) domain protein	90	90	89	90	89	100	100	100	90
OEM_08040	amidohydrolase	91	91	91	90	90	100	100	100	85
OEM_08050	amidohydrolase	87	87	87	87	89	100	100	100	84
OEM_08060	putative 3-hydroxyisobutyrate dehydrogenase	81	81	81	82	81	100	100	100	81
OEM_08070	4-carboxymuconolactone decarboxylase	86	87	87	87	87	100	100	100	ND
OEM_08080	putative acyl-CoA dehydrogenase	87	87	87	86	86	100	100	100	86
OEM_08090	acyl-CoA dehydrogenase	85	85	85	85	90	100	100	100	87
OEM_08100	twin-arginine translocation pathway signal	85	84	85	85	83	100	100	100	87
OEM_08110	ISRSO5-transposase transposase	ND	ND	ND	ND	ND	100	100	100	ND
OEM_08120	hypothetical protein	85	85	85	85	86	100	100	100	ND
OEM_08130	hypothetical protein	87	87	87	87	87	100	100	100	81
OEM_08140	cytochrome P450 family protein	85	85	85	85	85	100	100	100	85

OEM_08150	hypothetical protein	89	89	89	87	88	100	100	100	90
OEM_08160	acetoacetyl-CoA reductase	85	85	85	86	85	100	100	100	87
OEM_08170	aldehyde dehydrogenase	88	88	88	88	86	100	100	99	87
OEM_08180	formyl-CoA transferase	88	88	88	88	87	100	100	100	88
OEM_08190	hypothetical protein	ND	ND	ND	ND	ND	100	100	ND	ND
OEM_08200	hypothetical protein	84	84	84	84	81	100	100	100	82
OEM_08210	ABC superfamily ATP binding cassette transporter, membrane protein	84	84	83	83	83	100	100	99	81
OEM_08220	virulence factor mce family protein	72	72	72	72	72	100	100	99	70
OEM_08230	virulence factor mce family protein	75	75	75	75	76	100	100	100	76
OEM_08240	virulence factor mce family protein	73	73	73	73	71	100	100	100	72
OEM_08250	virulence factor mce family protein	73	73	73	73	73	100	100	100	74
OEM_08260	virulence factor mce family protein	76	76	76	76	75	100	100	100	ND
OEM_08270	virulence factor mce family protein	73	73	73	72	73	100	100	99	74

OEM_08280	hypothetical protein	ND	ND	ND	ND	ND	100	100	100	ND
OEM_08290	twin-arginine translocation pathway signal	67	ND	ND	ND	68	100	100	100	ND
OEM_08300	hypothetical protein	65	65	65	64	ND	100	100	98	67
OEM_08310	hypothetical protein	73	73	73	74	ND	100	100	100	ND
OEM_08320	ISMsm2 transposase	ND	ND	ND	ND	ND	100	100	100	ND
OEM_08330	TetR family transcriptional regulator	70	70	71	71	72	100	100	100	71
OEM_08340	hypothetical protein	84	84	84	84	84	100	100	100	84
OEM_08350	IS111A/IS1328/IS1533 transposase	ND	ND	ND	ND	ND	100	100	100	ND
OEM_08360	hypothetical protein	ND	ND	ND	ND	ND	100	100	100	ND
OEM_08370	hypothetical protein	85	85	85	86	85	100	100	100	84
OEM_08380	cytochrome c oxidase subunit III family protein	84	84	84	84	83	99	99	99	85
OEM_08390	hypothetical protein	76	76	76	77	74	100	100	100	79
OEM_08400	unspecific monooxygenase	84	84	84	84	85	100	100	100	85
OEM_08410	transcriptional regulator	83	83	83	81	82	100	100	100	81
OEM_08420	enoyl-CoA hydratase/isomerase	83	83	83	82	81	100	100	100	83

OEM_08430	hypothetical protein	72	72	72	72	72	100	100	100	72
OEM_08440	amidohydrolase	92	92	92	92	93	100	100	100	93
OEM_08450	acyl-CoA synthetase	86	86	86	86	85	100	100	100	86
OEM_08460	hypothetical protein	ND	ND	ND	ND	ND	100	100	100	ND
OEM_08470	twin-arginine translocation pathway signal	ND	ND	ND	ND	ND	100	100	100	ND
OEM_08480	FadD family protein	81	81	81	82	82	100	100	99	82
OEM_08490	BFD family (2Fe-2S)-binding region	84	84	84	84	84	100	100	100	81
OEM_08500	indolepyruvate decarboxylase	86	86	86	86	86	100	100	99	87
OEM_08510	cyclase/dehydrase	88	88	88	89	88	100	100	100	88
OEM_08520	integrase catalytic subunit	ND	ND	ND	ND	ND	100	100	ND	ND
OEM_08530	transposase, IS4 family protein	ND	ND	ND	ND	ND	100	100	ND	ND
OEM_08540	hypothetical protein	ND	ND	ND	ND	ND	100	100	ND	ND
OEM_08550	fatty-acid-CoA racemase	86	86	86	86	86	100	100	100	85
OEM_08560	cyclase/dehydrase	87	88	87	87	87	100	100	100	87
OEM_08570	cyclase/dehydrase	89	89	89	88	88	100	100	100	89
OEM_08580	putative cysteine-S-	86	86	86	85	86	100	100	100	85

	conjugate beta-lyase									
OEM_08590	fatty oxidation complex	93	92	93	93	93	100	100	95	93

[a] *M. yongonense* DSM 45126^T; [b] *M. intracellulare* ATCC 13950^T; [c] *M. parascrofulaceum* ATCC BAA-614^T; [d] *M. avium* 104

Supplementary Table S2. A locus containing non-mycobacterial genes in the *M. yongonense* Type I strains, but not in the *M. yongonense* Type II strains.

ORFs	Descriptions	Species	Nucleotide sequence similarities (%)
OEM_51290	enolase 2	*Nocardia nova*	69
OEM_51300	NADH/ubiquinone/plastoquinone (complex I)	*Nocardia nova*	67
OEM_51310	NADH ubiquinone oxidoreductase	*Nocardia nova*	72
OEM_51320	NADH-ubiquinone oxidoreductase	*Acidothermus cellulolyticus*	66
OEM_51330	NADH dehydrogenase	*Nocardia nova*	74
OEM_51340	hydrogenase subunit	*Nocardia nova*	71
OEM_51350	respiratory-chain NADH dehydrogenase, subunit 1	*Nocardia nova*	72
OEM_51360	hypothetical protein	-	-
OEM_51370	hypothetical protein	*Mycobacterium marinum*	74
OEM_51380	hypothetical protein	-	-
OEM_51390	hypothetical protein	-	-
OEM_51400	DNA mismatch repair protein MutS domain-containing protein	*Nocardia nova*	66
OEM_51410	DNA mismatch repair protein MutS domain protein	*Pseudomonas citronellolis*	68

Table S3. Additional list of NCBI accession numbers of *MutS4* homologs from prokaryotic species

Taxonomic group	Species Name	MutS4A	MutS4B
Eubacteria			
Actinobacteria	*Acidothermus cellulolyticus*	WP_011719570.1	WP_011719571.1
Actinobacteria	*Amycolatopsis orientalis*	WP_037359858.1	WP_037359860.1
Actinobacteria	*Alloactinosynnema iranicum*	-	SDD11334.1
Actinobacteria	*Arthrobacter* sp.	OEH63335.1	WP_083262057.1
Actinobacteria	*Nocardia nova*	-	WP_025351135.1
Actinobacteria	*Rhodococcus* sp.	WP_064074701.1	WP_064074690.1
Actinobacteria	*Rubrobacter radiotolerans*	AHY48378.1	WP_051590080.1
Actinobacteria	*Streptomyces alni*	SFE47000.1	SFE46975.1
Actinobacteria	*Mycobacterium branderi*	WP_083134047.1	WP_083134046.1
Actinobacteria	*Mycobacterium colombiense*	WP_076102547.1	WP_076102436.1
Actinobacteria	*Mycobacterium heckeshornense*	WP_048893703.1	WP_048893702.1
Actinobacteria	*Mycobacterium heidelbergense*	WP_083073024.1	WP_083073023.1
Actinobacteria	*Mycobacterium heraklionense*	WP_047319267.1	WP_047319268.1
Actinobacteria	*Mycobacterium icosiumassiliensis*	WP_067968125.1	WP_067967705.1
Actinobacteria	*Mycobacterium intermedium*	WP_069418183.1	WP_069418184.1
Actinobacteria	*Mycobacterium kyorinense*	WP_045385556.1	WP_045385553.1
Actinobacteria	*Mycobacterium malmoense*	WP_083008849.1	WP_083008852.1
Actinobacteria	*Mycobacterium sherrisii*	WP_069401098.1	WP_069401099.1
Actinobacteria	*Mycobacterium xenopi*	WP_085193923.1	WP_085193921.1
Actinobacteria	*Mycobacterium* sp. 141	WP_019971131.1	WP_019971130.1
Actinobacteria	*Mycobacterium* sp. 155	WP_026256085.1	WP_018600051.1
Actinobacteria	*Mycobacterium* sp. TKK-01-0059	KEF98747.1	KEF98748.1
Bacteroidetes	*Niastella vici*	-	WP_081153523.1
Chloroflexi	*Anaerolinea thermolimosa*	WP_062192114.1	WP_062192112.1
Chloroflexi	*Caldilinea aerophila*	WP_014433385.1	BAM00150.1
Chloroflexi	*Chloroflexus aurantiacus*	YP_001633979.1	YP_001633980.1
Chloroflexi	*Leptolinea tardivitalis*	WP_062422624.1	WP_062422625.1
Chloroflexi	*Ornatilinea apprima*	WP_075062663.1	WP_075062662.1
Chloroflexi	*Thermanaerothrix daxensis*	WP_054521131.1	WP_054521132.1
Chloroflexi	*Thermogemmatispora carboxidivorans*	WP_052889587.1	WP_052889586.1
Deinococcus-Thermus	*Meiothermus silvanus*	WP_013159152.1	WP_013159153.1
Firmicutes	*Alicyclobacillus shizuokensis*	-	WP_067929216.1
Firmicutes	*Caldanaerovirga acetigignens*	WP_073256691.1	WP_073256688.1
Firmicutes	*Clostridium* sp.	WP_066647536.1	WP_066647535.1
Firmicutes	*Halanaerobium hydrogeniformans*	WP_013405794.1	WP_013405793.1
Firmicutes	*Moorella glycerini*	WP_054937233.1	WP_054937232.1
Firmicutes	*Propionispora vibrioides*	SEP01060.1	-

Firmicutes	*Thermoanaerobacter* sp.	WP_003869704.1	WP_004398856.1
Firmicutes	*Thermoanaerobacterium xylanolyticum*	WP_013787394.1	WP_013787395.1
Firmicutes	*Thermovenabulum gondwanense*	WP_068748666.1	WP_068748667.1
α-proteobacteria	*Chelatococcus daeguensis*	WP_063186961.1	WP_082831592.1
α-proteobacteria	*Rhizobium leguminosarum*	WP_080725538.1	WP_037063550.1
α-proteobacteria	*Ochrobactrum anthropi*	KXO73093.1	-
α-proteobacteria	*Nitrobacter* sp.	OJU26665.1	-
α-proteobacteria	*Elioraea tepidiphila*	WP_084596595.1	WP_019015166.1
α-proteobacteria	*Methylobacterium extorquens*	WP_015822420.1	WP_015822421.1
β-proteobacteria	*Achromobacter denitrificans*	WP_082775779.1	WP_082775778.1
β-proteobacteria	*Caballeronia sordidicola*	WP_031360372.1	WP_031360373.1
β-proteobacteria	*Candidimonas bauzanensis*	WP_073104217.1	WP_073104219.1
β-proteobacteria	*Castellaniella caeni*	WP_084386561.1	WP_066455858.1
β-proteobacteria	*Pandoraea apista*	-	WP_042118694.1
β-proteobacteria	*Paraburkholderia acidipaludis*	WP_027796032.1	WP_043202652.1
δ-proteobacteria	*Desulfacinum hydrothermale*	WP_084057625.1	WP_084057624.1
δ-proteobacteria	*Desulfatirhabdium butyrativorans*	WP_028323924.1	WP_051327939.1
γ-proteobacteria	*Dyella jiangningensis*	WP_083337459.1	WP_074546061.1
γ-proteobacteria	*Frateuria* sp.	WP_056007358.1	WP_056007355.1
γ-proteobacteria	*Methylobacter tundripaludum*	WP_031436729.1	WP_031436728.1
γ-proteobacteria	*Pseudomonas citronellolis*	ANI18508.1	WP_064583374.1
γ-proteobacteria	*Rhodanobacter* sp.	WP_051257556.1	WP_051257557.1
Thermotogae	*Mesoaciditoga lauensis*	WP_036222510.1	WP_036222432.1
Verrucomicrobia	*Chthoniobacter flavus*	WP_006980653.1	WP_006980652.1
Archea			
Euryarchaeota	*Methanocella paludicola*	BAI62550.1	BAI62551.1
Euryarchaeota	*Ferroplasma acidarmanus*	WP_009887857.1	WP_009887856.1
Euryarchaeota	*Picrophilus oshimae*	WP_084272695.1	WP_084272696.1
Euryarchaeota	*Thermoplasma volcanium*	WP_010916932.1	WP_010916933.1

Supplementary Table S4. Comparison the numbers of sequenced colonies and average length of putative homologous recombination sequences in the transformed recombinant *M. smegmatis* strains.

	rSmeg-pMV306-p317	rSmeg-D6-p317	*P*-value[a]
Recombination occurred /Total numbers (%)	5/16 (31.3 %)	27/36 (75 %)	0.020
Average length	44.0	121.3	< 0.001

[a] *P*-value was calculated by Chi-Square and student-*t* tests.
